# Breast Tumors with Elevated Expression of 1q Candidate Genes Confer Poor Clinical Outcome and Sensitivity to Ras/PI3K Inhibition

**DOI:** 10.1371/journal.pone.0077553

**Published:** 2013-10-17

**Authors:** Muthulakshmi Muthuswami, Vignesh Ramesh, Saikat Banerjee, Soundara Viveka Thangaraj, Jayaprakash Periasamy, Divya Bhaskar Rao, Georgina D. Barnabas, Swetha Raghavan, Kumaresan Ganesan

**Affiliations:** 1 Cancer Genetics Laboratory, Department of Genetics, Centre for Excellence in Genomic Sciences, School of Biological Sciences, Madurai Kamaraj University, Madurai, India; 2 Department of Biotechnology, Indian Institute of Technology Madras, Chenna, India; Mayo Clinic, United States of America

## Abstract

Genomic aberrations are common in cancers and the long arm of chromosome 1 is known for its frequent amplifications in breast cancer. However, the key candidate genes of 1q, and their contribution in breast cancer pathogenesis remain unexplored. We have analyzed the gene expression profiles of 1635 breast tumor samples using meta-analysis based approach and identified clinically significant candidates from chromosome 1q. Seven candidate genes including exonuclease 1 (*EXO1*) are consistently over expressed in breast tumors, specifically in high grade and aggressive breast tumors with poor clinical outcome. We derived a *EXO1* co-expression module from the mRNA profiles of breast tumors which comprises 1q candidate genes and their co-expressed genes. By integrative functional genomics investigation, we identified the involvement of EGFR, RAS, PI3K / AKT, MYC, E2F signaling in the regulation of these selected 1q genes in breast tumors and breast cancer cell lines. Expression of *EXO1* module was found as indicative of elevated cell proliferation, genomic instability, activated RAS/AKT/MYC/E2F1 signaling pathways and loss of p53 activity in breast tumors. mRNA–drug connectivity analysis indicates inhibition of RAS/PI3K as a possible targeted therapeutic approach for the patients with activated *EXO1* module in breast tumors. Thus, we identified seven 1q candidate genes strongly associated with the poor survival of breast cancer patients and identified the possibility of targeting them with EGFR/RAS/PI3K inhibitors.

## Introduction

Breast cancer is one of the most common malignancies in women worldwide. It is also one of the well explored human cancers with genome-wide technologies. In the past two decades, a number of breast cancer genomics investigations contributed to the understanding of the molecular portfolio of breast cancers [1,2]. Several cancer genes and gene signatures indicative of breast cancer sub-type, progression, prognosis, and disease aggressiveness have been derived from mRNA profiles of breast tumors [3,4]. Accumulating genome-wide profiles of various tumors in microarray repositories have revolutionized the field of cancer biology owing to their continuous contribution in addressing various questions in basic and translational research through meta-analysis based genomics approaches [5,6]. This possibility of dissecting and integrating cancer genomics and transcriptomics data in several possible contexts paved ways for identification of novel cancer biomarkers and to uncover various mechanisms involved in the process of carcinogenesis.

Genomic aberrations are the hallmarks of cancer genomes and breast cancer genomes have been characterized for copy number variations and associated biological and pathological features [7,8]. Prevalence of several genomic amplifications (1q, 8q, 17q, 20q) and deletions (5q, 16q, 8p) in breast cancers reflect the definite involvement of specific molecular factors of those loci and associated processes that contribute in cancer development [9]. Aberrations in chromosome 1 are more frequent in various cancers [10]. The long arm of chromosome 1 (1q) is known for its frequent copy number gains whereas 1p region often shows copy number loss [11]. The most interesting aspect of 1q gain in breast cancer is its prevalence in almost all types of breast cancer like Estrogen Receptor (ER) positive, ER negative [12], Luminal A [13], Ductal carcinoma in situ (DCIS) and Invasive ductal carcinoma (IDC) [14]. Recurrent 1q gain in breast cancers [11,15], and combined investigations of chromosome 1q gain with other amplifications have been reported [16,17]. Since 1q gain comprises several hundreds of genes, the functional consequences of this recurrent gain remains hard to establish [18]. The potential 1q candidate genes and their specific contribution in breast cancer development remain un-identified. Therefore, in this study, we systematically examined the clinical significance of the expression of all 1q genes in breast tumors by meta analysis based integrative genomics approach and identified 7 potential candidate target genes. Motivated by the occurrence of underexplored candidacy of *EXO1* from 1q, we investigated the upstream regulatory pathways and expression pattern across breast cancer sub-types. Further, consensus *EXO1* co-expressing gene set was derived and is predicative of biological, clinical and pathological features of breast tumors. We also identified a possible therapeutic targeting approach for breast tumors with elevated *EXO1* modular expression.

## Materials and Methods

### Data pre-processing

Datasets used in the study were collected from original references or microarray repositories Gene Expression Omnibus (GEO), ArrayExpress, *etc*. The expression profiles taken for the study were normalized while necessary and the expression values were log_2_ transformed in the case of single channel data and log ratio data from dual channel data was used as such. The probes were mapped to unique gene symbols with appropriate Affymetrix or Agilent annotation files. The expression values of genes with multiple probes were averaged and used for downstream analysis. 

### Survival analysis

We considered relapse free survival and overall survival information of the breast cancer patient cohorts for predicting the clinical outcome. Hazard Ratios (HR) and significant p-values were calculated independently for each dataset using coxph function of Survival R package [19,20]. Genes with p-value < 0.005 from Wald statistic were considered significant. Combined HRs for each gene was estimated using inverse variance-weighted method with random effects model. Univariate and Multivariate Cox proportional hazards model for/with other clinical covariates was performed using Rcmdr package [21]. Based on median value of *EXO1* gene expression, the samples were stratified into two *EXO1*+ve and *EXO1*-ve groups, and are then used as one of the variables for univariate and multivariate analysis.

Kaplan Meier estimate was used for plotting survival curves and p-values were calculated using log-rank test. In case for the *EXO1* module, average gene expression values were used for computing survival curve.

### Data analysis

*EXO1* gene expression values were extracted from normalized log_2_ transformed breast tumor profiles. The significant difference in gene expression between any two groups of breast tumor samples were calculated using Student’s t-test (two tailed) and while calculating for more than 2 groups (i.e. for grade), ANOVA was performed.

For defining *EXO1* module, the Pearson correlation measure was calculated for each gene – *EXO1* pair independently for all the datasets. With an assumption that effect sizes derived from correlation coefficients vary from dataset to dataset, we used random effects model for deriving the weighted average from correlation coefficients of individual datasets. A stringent cut-off of 0.6 and above with p-value<0.001 was fixed in defining the *EXO1* module genes. Ontological terms for module genes were given based on DAVID function annotation tool and Cytoscape was used for network visualization [22].

Principal component analysis (PCA) was applied using Rcmdr package from CRAN. Transcription factor binding site analysis for single gene was performed using MAPPER database and for geneset DIRE tool was used. Significant over representation of *EXO1* module genes to breast cancer signatures was estimated using hypergeometric distribution function.

### Pathway activation analysis

Gene signatures representative of particular phenotype/condition were collected from MsigDB (http://www.broadinstitute.org/gsea/msigdb/genesets.jsp?collection=CGP) or from the original references. Detailed descriptions of the signatures and their sources were provided with Table S3. Each signature represented by corresponding up and down tags were scanned against the gene expression profiles of breast cancer profiles as mentioned earlier [23]. 

### Cell culture

MCF7, T47-D, ZR75, MDA-MB-231, MDA-MB-453, MDA-MB-468, HBL-100 and AGS cells were obtained from National Centre for Cell Sciences (NCCS), Pune, India. Hs578T, Kato III and SKBR-3 were from American Type Culture Collection (ATCC), Manassas, USA. YCC16 cell line was from Yonsei Cancer Centre, Korea [24]. The cells were cultured in the specified media (HiMedia); MCF-7 and YCC16: Minimal Essential Media( MEM), T47-D, ZR 75 & Kato III: RPMI 1640, Hs 578T: DMEM, SK-BR-3 & HBL-100: McCoy’s 5a medium, MDA-MB-231, MDA-MB-453 and MDA-MB-468: Leibovitz, AGS: DMEM-F12, with the supplements L-glutamine (2 mM), sodium pyruvate (1 mM), sodium bicarbonate (1.5 g/L), non-essential amino acids (0.1 mM), penicillin (100 μg/ml), streptomycin (100 μg/ml) (HiMedia) and 10% foetal bovine serum (20 % for Kato III) (Sigma).

### Drug Treatment, RT-PCR & Western Blotting

For drug exposure experiments 5x10^5^ cells/well were seeded in a 6-well cell culture dishes. All drug treatments were done upon cells reaching 80 % confluency. For silymarin treatment, the cells were grown in serum-free media for 24 hours prior to drug treatment and treated with 50 μM, 100 μM and 200 μM concentrations. Salirasib was treated at three different concentrations 25 μM, 75 μM and 150 μM. Alkylating agents carboplatin (5, 10 and 15 μg/ml), Cyclophosphamide (2.5, 5 and 10 μM) and Ifosfamide (0.01, 0.05 and 0.1 μg/ml) were used for the treatment in MCF-7 cell lines. All drug treatments were performed for 24 hours and the total RNA was isolated by Trizol (Invitrogen) as per the manufacturer’s protocol. 2 μg of RNA was used to synthesize cDNA using reverse transcriptase (Invitrogen) and the cDNA was used for semi-quantitative PCR analysis. Comparative analysis of the relative expression of *EXO1* protein across the panel of breast cancer cell lysates was performed by standard Western blotting using Anti-*EXO1* Antibody (LSBIO, LS-B3818 and Sigma, WH0009156M1). 30 μg whole cell lysate were resolved in 12 % gel and 1:100 dilution (LSBIO, LS-B3818) or 1:500 dilution (Sigma, WH0009156M1) of *EXO1* antibody was used. Vinculin was probed as loading control for the blotting.

### Luciferase Reporter Assays

For luciferase reporter experiments, 75,000 cells/well were seeded in 24-well cell culture dishes. After 24 hours, E2F, MYC, FOXO3 firefly reporter plasmids along with renilla reporter plasmid (SA Biosciences) as internal control in a ratio of 40:1 were transfected using Fugene transfection reagent (Promega). The transfection was performed as per the manufacturer’s instructions. In case of promoter activity assay, E2F or Myc ORF plasmid (Addgene) and *EXO1* promoter reporter in the ratio of 1:1 along with renilla reporter plasmid (40:1) was used for transfection. After 48 hours of transfection, the cells were harvested and the luciferase activity was measured using dual luciferase assay protocol [25] in SpectraMax L (Molecular Devices). The normalization was performed by dividing the firefly reporter value by renilla reporter value to obtain relative luciferase activity. Fold change was obtained by dividing the normalized values of the respective pathway reporters with the negative control reporter. Student’s t-test (two tailed) was used for the analysis of significance.

## Results

### Identification of 1q genes predictive of poor survival in breast tumors

**Figure 1:**
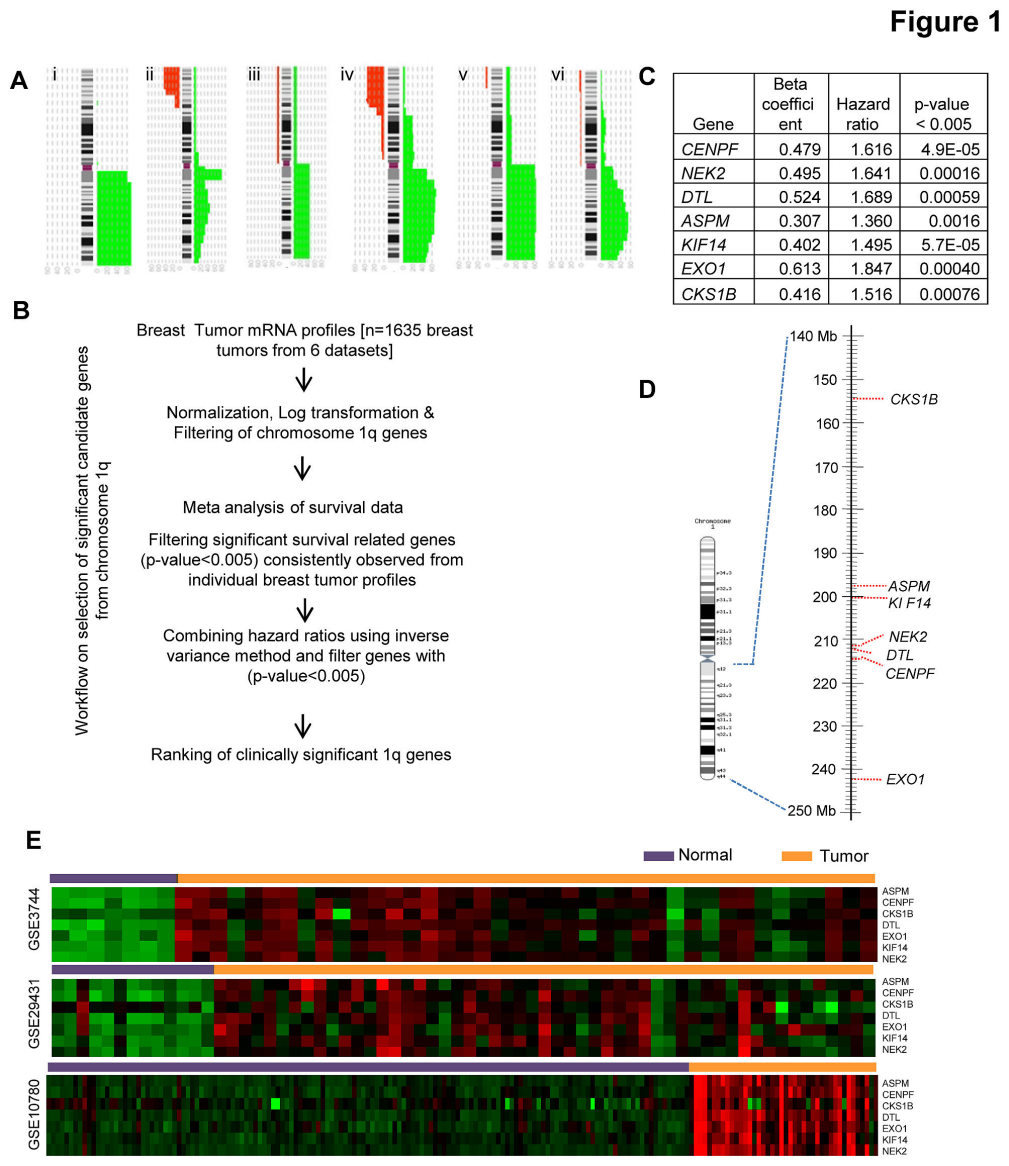
Identification of genes associated with poor clinical outcome in 1q amplicon of breast tumors. (A) High frequency amplification of 1q region in multiple cohorts of breast tumors. Graphs I – VI represent inferred copy-number of probes at chromosome 1 from the aCGH studies [61-66]. These graphical representations were the outcome of data visualized through Progenetix [67,68]. Copy number gain and loss are represented with green and red colors respectively. In all these five cohorts, 1q is amplified in 40 - 60 % of tumors. (B) Meta analysis – workflow for the identification 1q genes associated with patient survival. (C) Cox regression coefficient, hazard ratio and p-value of the shortlisted 1q genes, (D) Chromosomal map showing the locations of clinically significant 1q genes. (E) The identified 1q candidate genes showing an elevated expression in breast tumors when compared to the non-cancerous breast tissues in 3 different cohorts.

Searching for prevalent genomic amplifications in breast cancers with the genome-wide copy number profiles in progenetix database has shown gain in chromosome 1q as prevalent and occurs in 40 - 50 % of breast cancer patients in multiple cohorts (Figure 1A). Recurrent copy number gain regions in cancer genomes often harbour genes that facilitate tumor development and progression. Despite being the most frequent chromosomal amplification in breast cancer, the candidate cancer genes of 1q amplicon have not been systematically analyzed. By a systematic integrative genomics analysis work-flow (Figure 1B), based on the gene’s association with the survival of breast cancer patients, we short-listed the clinically significant 1q candidate genes. Totally, mRNA expression profiles of 1635 breast tumor samples from 6 independent studies were investigated (Table S1). Cox regression analysis of 498 genes from 1q locus primarily filtered 10 genes that are consistently associated with survival of the patients in at least 3 of the 6 cohorts with the p-value <0.005. The subsequent filtering with the combined p-value <0.005 across 6 datasets, yielded 7 candidate genes *ASPM*, *KIF14*, *NEK2*, *DTL*, *CENPF*, *CKS1B* and *EXO1* (Figure 1C) that are significantly associated with poor clinical outcome of the breast cancer patients (Table S2). Since, the physical neighbour genes of a chromosomal locus would sometime have coordinated pattern of expression [26], we mapped the candidate genes to their corresponding genomic location in chromosome 1q and the mapping identified that these genes are not tightly clustered at a specific locus in 1q (Figure 1D). Investigation of the expression pattern of these candidate genes reveals their elevated expression in breast tumor samples when compared to normal breast tissues (Figure 1E). Thus, the frequent 1q amplification, consistent elevated expression of seven 1q genes and their association with poor survival suggests their involvement in breast cancers.

### Elevated *EXO1* expression is associated with poor clinical outcome in breast cancer

**Figure 2:**
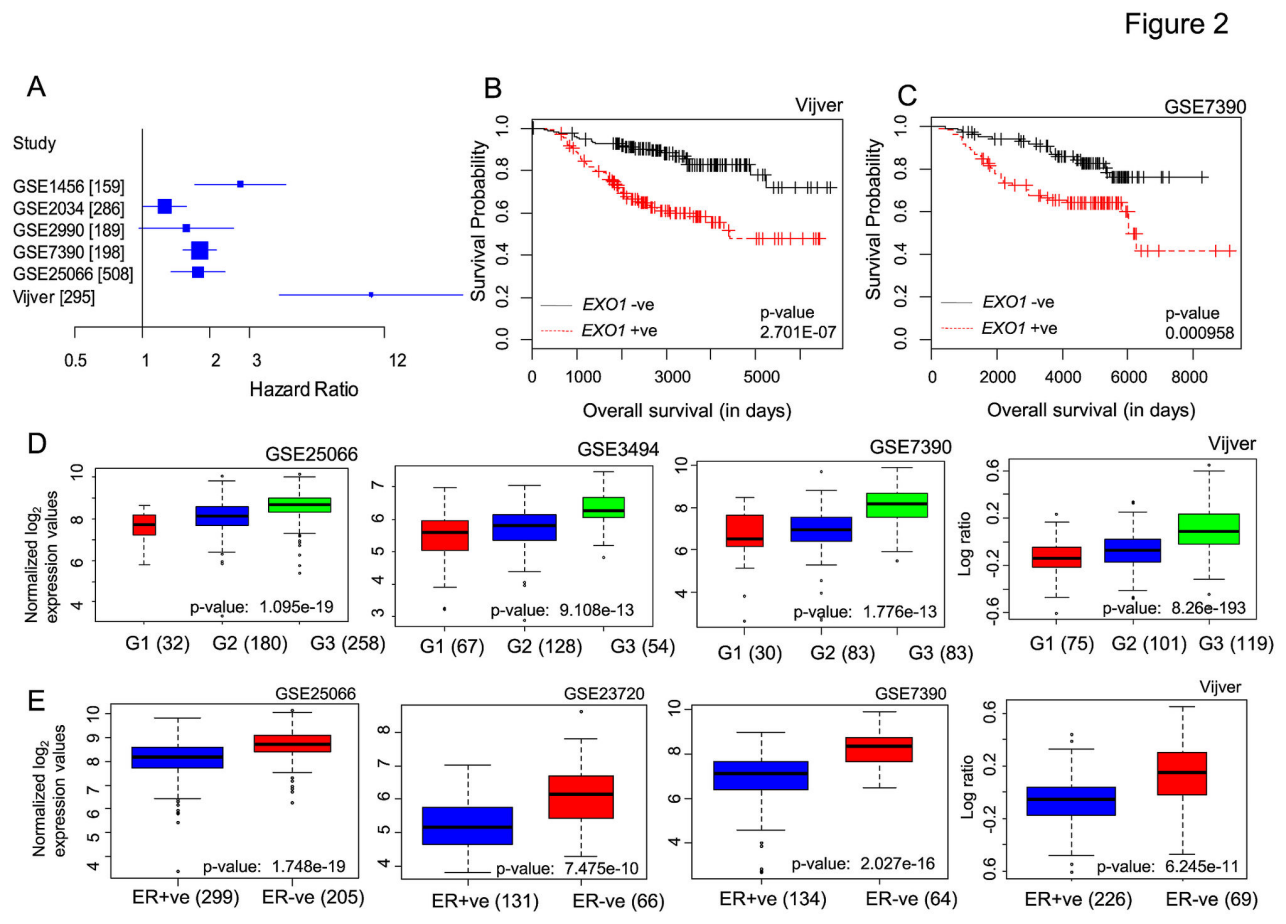
*EXO1* exhibits elevated expression in high grade and ER negative breast cancers. (A) Hazard ratio for *EXO1* gene expression with relapse free survival as end point in 6 different breast tumor datasets shown as forest plot. B & C) Kaplan Meier curve based on *EXO1* gene expression with overall survival as end point in breast tumor profiles, Vijver and GSE7390 respectively. The log-rank test was used for computing significance level for the survival curves. (D) and (E) Comparative analysis of *EXO1* gene expression across different grades and subgroups of breast tumors reveal elevated *EXO1* expression in high grade (D) and ER negative subgroups of breast tumors (E). The total number of samples in each group is denoted in parenthesis and the GEO accessions of the profiles are shown on the top of each boxplot.

While the breast cancer candidacy of *CENPF, KIF14, NEK2, DTL, CKS1B, ASPM* and *EXO1* genes have been identified earlier [27-33], there is only one prominent report indicating the candidacy of *EXO1* in breast cancers [33] and remains to be investigated. Apart from the reported polymorphisms [34-36] and elevated expression of *EXO1* in Ductal carcinoma *in situ* [33], also there are contradicting reports relating *EXO1* loss of function to increased susceptibility to lymphomas [37], urging the need for further investigation. Therefore, the current identification of the consistently elevated expression of *EXO1* gene and its association with poor survival in breast cancers in multiple co-horts is a significant observation. Apart from the identified association between *EXO1* expression and poor survival, we further confirmed its clear association with poor clinical outcome using overall survival as end point. Hazard ratio of *EXO1* gene expression across 6 independent breast tumor profiles with relapse free survival as endpoint (Figure 2A) and Kaplan Meier survival curves for *EXO1* expression with overall survival information as endpoint (Figure 2B-2C) clearly implies the poor clinical outcome associated with higher level *EXO1* expression in breast cancer patients. Further, univariate and multivariate Cox proportional hazards model was performed in 3 breast tumor profiles for which maximal clinical variables were available. Univariate analysis revealed the significant association of *EXO1* expression with relapse of breast tumors comparable to other individual clinical variables such as age, tumor size, grade, ER status, and lymph node status. In multivariate analysis also *EXO1* retained the statistical significance with p-value <0.05 in all 3 analyzed profiles (Table S3). This indicates *EXO1* to be an independent predictor of survival, and needs to be investigated in larger cohorts. Since *EXO1* expression is associated with the poor prognosis, there arises a question on *EXO1* gene expression in the context of specific subtype(s)/group(s) of breast cancers. We addressed this by investigating the expression of *EXO1* in 18 breast cancer transcriptome profiles that are available from microarray repositories GEO and Array Express (Table S1). Systematic analysis of *EXO1* expression across these breast cancer profiles revealed consistent elevated expression of *EXO1* in i) breast tumors when compared to normal breast tissues (Figure S1A), ii) higher grade breast tumors (Figure 2D), iii) ER negative while compared to ER positive tumors (Figure 2E), iv) PR negative subtype of breast tumors (Figure S1B), and v) basal subtype breast tumors when compared to luminal subtypes (Figure S2). We also found subtle elevation of *EXO1* expression in invasive ductal breast carcinoma and metastatic breast tumors with modest statistical significance (Figure S1C-S1D). Thus, across multiple cohorts of breast tumors from various populations that were profiled across various microarray platforms, we observed highly consistent and elevated expression of *EXO1* in high grade, basal, ER negative and PR negative subtypes. All these show a strong association between *EXO1* expression and poor clinical outcome in breast cancer patients. 

### Identification of the possible upstream regulators of *EXO1* in Breast tumors

**Figure 3:**
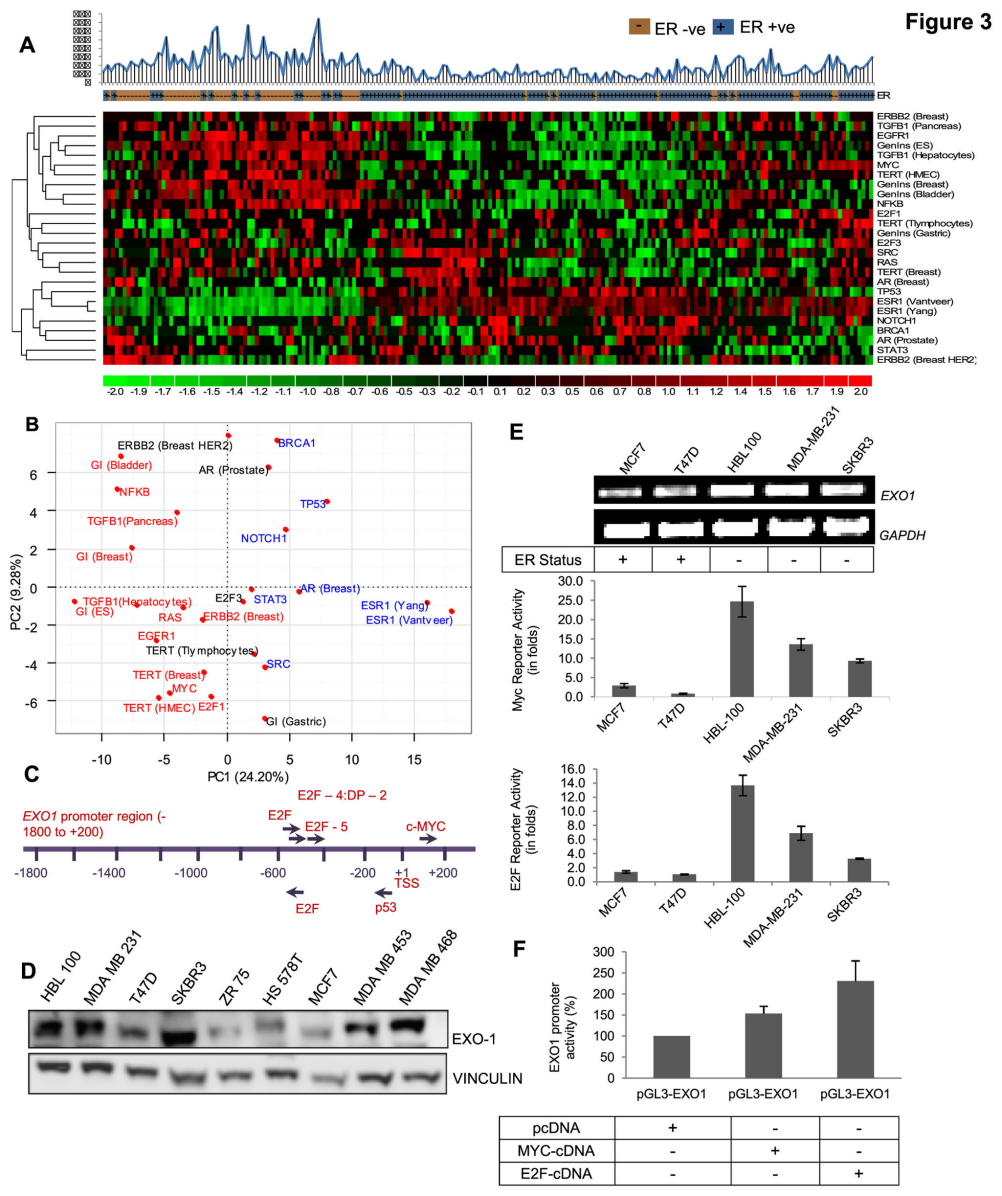
Identification of signaling processes associated with *EXO1* expression in breast tumors. (A) The activation status of oncogenic signaling pathways and other cancer signatures in 198 breast tumor samples shown as heatmap. Different pathway or cellular process specific gene-sets were analyzed and the comprehensive activation (red) and inactivation (green) pattern across samples is shown. (B) Principal component analysis of the pathway activation pattern in breast tumors shown in Figure A. (C) Occurrence of E2F, Myc and p53 transcription factor binding sites in *EXO1* promoter. (D) Western blot showing the expression of *EXO1* in a panel of breast cancer cell lines. (E) RT-PCR showing the expression of *EXO1* in a panel of breast cancer cell lines (top panel). *In* *vitro* pathway specific reporter assays showing relative activation of Myc and E2F transcription factors in three of the higher level *EXO1* expressing cell lines (ER –ve) while compared to two lower level *EXO1* expressing cell lines (ER +ve) (Lower panel). These assays have been performed thrice with triplicates. (F) Luciferase reporter assay showing the activation of *EXO1* promoter upon the transfection of E2F1 or c-Myc cDNA in MDA-MB231 cells (p-values were 0.013 and 0.009 respectively). pGL3-*EXO1* is *EXO1* promoter construct in pGL3 enhancer reporter vector. The experiment has been performed thrice with triplicates.

Elevated expression of *EXO1* in breast cancer patients with poor clinical outcome indicates the need for investigating the pathways and factors regulating *EXO1* expression. In order to identify the possible upstream regulators of *EXO1*, gene signature based pathway activation pattern was investigated in the mRNA expression profiles of breast cancer samples. 26 signatures or gene sets representing various pathways and molecular cellular processes were collected from MsigDB and original literatures and used for this analysis (Table S4). Two breast cancer profiles representative of breast tumors [GSE7930], and breast cancer cell lines [E-TABM-157] were analyzed to score the activation of pathway signatures using *in-silico* gene-set based pathway activation prediction approach described earlier [23,38]. Hierarchical clustering (Figure 3A and Figure S3A), regression analysis (Table S5-S6) and principal component analysis (Figure 3B and Figure S3B) of signature based activation pattern of pathways revealed a significant positive association of *EXO1* gene expression with the activation of MYC, RAS, EGFR, Genomic instability, and E2F pathways in breast tumors and cell lines. On the other hand, Estrogen Receptor (ESR1), p53, and BRCA pathways showed negative association with *EXO1* expression (Figure 3A - 3B, Figure S3, Table S5-S6). This shows that EGFR, RAS, MYC and E2F1 could be the possible upstream regulators of *EXO1.*

### *EXO1* expression is regulated by Myc & E2F transcription factors

In order to identify suitable cell lines for the *in vitro* experiments, expression of *EXO1* across a panel of breast cancer cell lines was probed by western blotting and RT-PCR. Overall, there is a concordance in *EXO1* expression pattern identified by Western blotting, RT-PCR and available microarray data (Figure 3D, 3E and Figure S4). Multiple associations indicate the possible regulation of *EXO1* by EGFR, RAS, MYC, and E2F1. First, the analysis of transcription factor binding sites in *EXO1* promoter region revealed the presence of MYC and E2F1 binding sites (Figure 3C). Second, we investigated the association between *EXO1* expression and inherent signalling / transcription factor activity of MYC and E2F transcription factors in a panel of breast cancer cell lines comprising few higher level *EXO1* expressing cells (HBL100, SKBR3 and MDA-MB-231) and a couple of lower level *EXO1* expressing cells (MCF7 and T47D) by *in vitro* reporter assay (Figure 3E). In five different breast cancer cell lines, the MYC and E2F reporter plasmids were transfected and assayed for the inherent un-induced transcriptional activity. The normalized luciferase reporter assay result shows a positive association between MYC and E2F reporter activity and *EXO1* expression in these cell lines with 5-20 folds higher activation of MYC and E2F in majority of *EXO1* expressing cell lines (Figure 3E).

**Figure 4:**
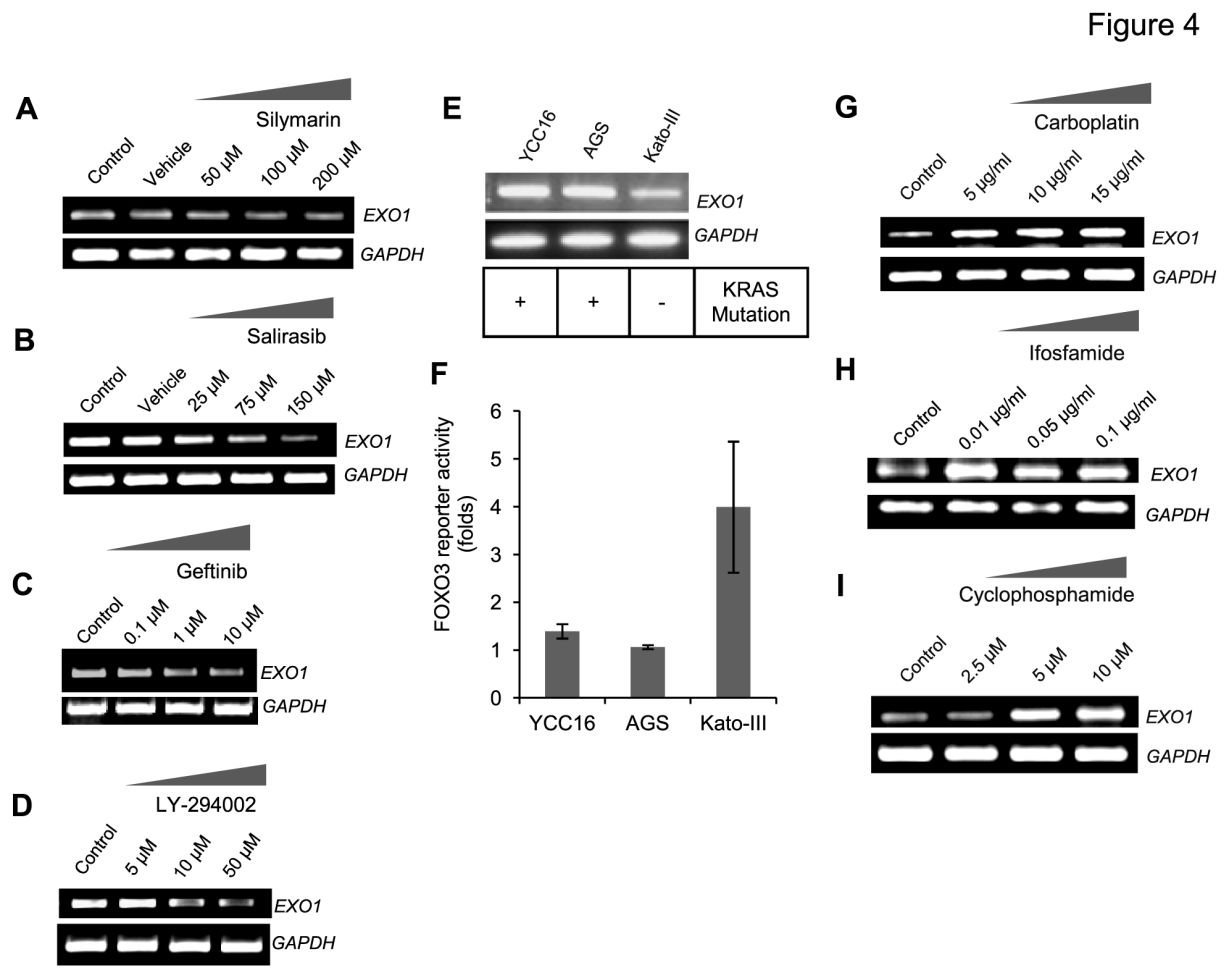
*In* *vitro* evaluation of Ras, EGFR, and PI3K/AKT pathway mediated regulation of *EXO1* expression. (A - D) Reduced expression of *EXO1* in breast cancer cells upon chemical inhibition of E2F, Ras, EGFR, and PI3K/AKT pathways using the specific inhibitors Silymarin, (A) Salirasib (B), Geftinib (C), and LY-294002 (D) respectively. While E2F & Ras inhibition are from MB-231 cells (A-B), PI3K/AKT & EGFR inhibition experiments (C-D) are from MB-468 cells. (E) RT-PCR showing that *EXO1* expression is relatively high in gastric cancer cell lines with KRAS mutation, while compared to the cell line without KRAS mutation. (F) FOXO3 reporter assay (inversely proportional to AKT pathway activity) showed relatively higher level activity of AKT in cell lines with higher level *EXO1* expression (YCC16 and AGS) compared to the cell line with relatively less folds of AKT activity (Kato III). (G - I) Elevated expression of *EXO1* in MCF7 cells upon exposure to DNA damaging agents carboplatin (G), Ifosfamide (H), and Cyclophosphamide (I). The luciferase assays have been performed thrice with triplicates. All semi-quantitative RT-PCRs were performed thrice.

To confirm the MYC and E2F mediated transcriptional regulation of *EXO1*, -2 kb promoter region of *EXO1* was cloned in pGL3-Enhancer reporter vector (designated as pGL3-*EXO1*). In MDA-MB-231 cells, pGL3-*EXO1* was transfected along with MYC and E2F cDNA (ORF cloned in mammalian expression vector) encoding plasmids and assayed for luciferase activity. The results implied a positive regulation of *EXO1* promoter by the transcription factors MYC and E2F1 (Figure 3F). Further, chemical inhibition of E2F activity by E2F inhibitor (Silymarin) reduced the expression of *EXO1* in MDA-MB-231 cell lines (Figure 4A). All these results demonstrate that MYC and E2F regulates the expression of *EXO1* in breast cancer cells.

### EGFR and RAS are the upstream regulators of *EXO1*

Apart from E2F and Myc, EGFR and RAS also showed positive association with the expression of *EXO1* in pathway pattern correlation analysis (Figure 3A - 3B). Therefore, we hypothesized RAS being the key downstream regulator of EGFR which in turn might govern the expression of *EXO1*. In order to investigate this association, we took a KRAS mutant breast cancer cell line, MDA-MB-231 and treated with RAS inhibitor, salirasib. This resulted in the inhibition of *EXO1* expression (Figure 4B). Subsequently to address the association between EGFR and *EXO1* gene expression, we treated EGFR positive breast cancer cell line, MDA-MB-468, with EGFR inhibitor, gefitinib. As expected, EGFR inhibition resulted in reduced expression of *EXO1* in MDA-MB-468, thereby illustrating the involvement of EGFR/RAS cascade in the regulation of *EXO1* gene expression in breast cancer cells (Figure 4C). The involvement of RAS/PI3K signalling cascade was confirmed owing to the reduction in *EXO1* expression upon treatment with PI3K/AKT inhibitor LY-294002 in the breast cancer cell line MDA-MB-468 (Figure 4D). We also found reduction in the expression of *EXO1* upon the same EGFR and PI3K/AKT inhibitor treatment in MDA-MB-231 and ZR-75 cells (data not shown). These show that *EXO1* is regulated through RAS/PI3K/AKT signalling in breast cancer cells. Interestingly, even in a panel comprising KRAS mutant and wild type gastric cancer cell lines, *EXO1* showed higher expression in KRAS mutant cell lines (YCC16 and AGS) when compared to wild type cells (KATOIII) (Figure 4E). Further, we analyzed the FOXO3 firefly luciferase reporter activity (a negative indicator of RAS/PI3K/AKT pathway activation) in the same gastric cancer cell lines and identified FOXO3 reporter activity to be higher in wild type cells (Kato III) while compared to KRAS mutant cell lines (AGS and YCC16) (Figure 4F). All these indicate that EGFR, Ras, PI3K, Myc, and E2F are involved in the regulation of *EXO1*. However, among these the direct and indirect regulations remain to be determined.

We also addressed the involvement of *EXO1* in DNA repair pathway in cancerous conditions since *EXO1* is known to play a vital role in DNA repair process by mismatch mediated repair mechanism [39]. This was addressed by treating MCF7 cell line with different alkylating agents carboplatin, cyclophosphamide and ifosfamide as they are well known to induce DNA repair [40-42]. The experiments showed *EXO1*’s increased expression with increasing concentration of these alkylating agents in a dose dependent manner thus supporting its role in DNA repair processes (Figure 4G-4I). Apart from DNA repair, another prime factor associated with *EXO1* expression is genomic instability (Figure 3A-3B). Since these alkylating agents also would elevate the genomic instability in cancer cells upon exposure [43], these results also indicate that *EXO1* expression is indicative of elevated genomic instability in cancer cells. 

### *EXO1* co-expressing genes are predictive of poor clinical outcome in breast cancer patients

**Figure 5:**
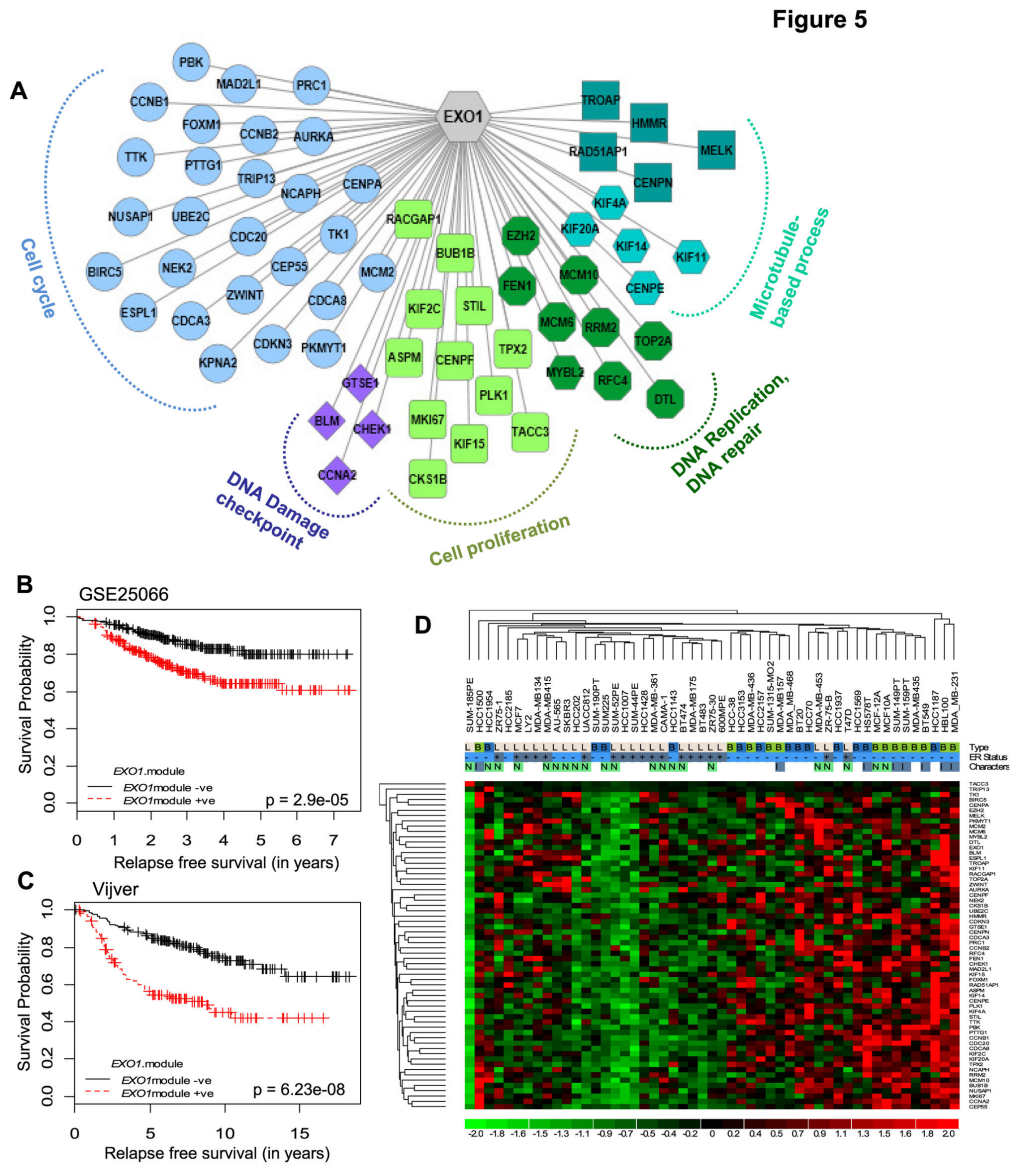
*EXO1* modular genes are tightly co-expressed and associated with poor prognosis in breast cancer patients : (A) Identification of *EXO1* modular genes using meta-analysis of correlation coefficients from multiple breast tumor datasets. Schematic *EXO1* centred network shows that *EXO1* co-expressed genes are involved in cell cycle and DNA repair. (B) and (C) Elevated expression of *EXO1* modular genes was associated with poor clinical outcome in breast tumor cohorts. (D) Heatmap showing the expression pattern of *EXO1* modular genes in 51 breast cancer cell lines. *EXO1* modular genes are highly expressed in aggressive and basal cell lines. I-Invasive, N-Non-invasive, B-Basal, L-Luminal and ER status of the cell lines are shown on the top.

Since we identified *EXO1* expression as indicative of poor prognosis and genomic instability with activated RAS/PI3K/AKT/MYC/E2F cascade, this observation needs to be explored for possible diagnostic and therapeutic implications. However, rather than single gene, a cluster of genes would be better predictors of a phenotype [44]. Therefore, we derived a set of genes which have the expression pattern very similar to *EXO1*. Since coexpressing genes would have the same pattern of gene regulation, correlation coefficients of genes coexpressing with *EXO1* were computed (Table S7) in 2479 samples from 9 different breast tumor transcriptome profiles (Table S1) and defined a module of genes that are tightly coexpressed with *EXO1* in breast tumors (Figure 5A). Further analysis on the features of *EXO1* modular genes mirrored *EXO1* in i) occurrence of enriched E2F binding sites in the promoter of *EXO1* modular genes (Figure S5) and ii) showing significant association with poor survival of the breast cancer patients (Figure 5B-5C). Another striking aspect of *EXO1* module is that it includes all seven 1q candidate genes that were originally identified as genes associated with poor survival in breast cancer patients. Analysis of the expression of *EXO1* modular genes in a comprehensive panel of 51 breast cancer cell lines showed higher expression in basal and invasive breast cancer cell lines (Figure 5D). *EXO1* modular genes showed higher expression in ER negative and higher grade breast tumors (Figure S6). However, a sub-set of ER positive tumors also showed elevated expression of *EXO1* modular genes. This shows that *EXO1* modular genes are capable of predicting aggressive breast tumors irrespective of ER status. 

### PI3K/AKT inhibition is the potential therapeutic strategy for *EXO1* module activated breast tumors

**Figure 6:**
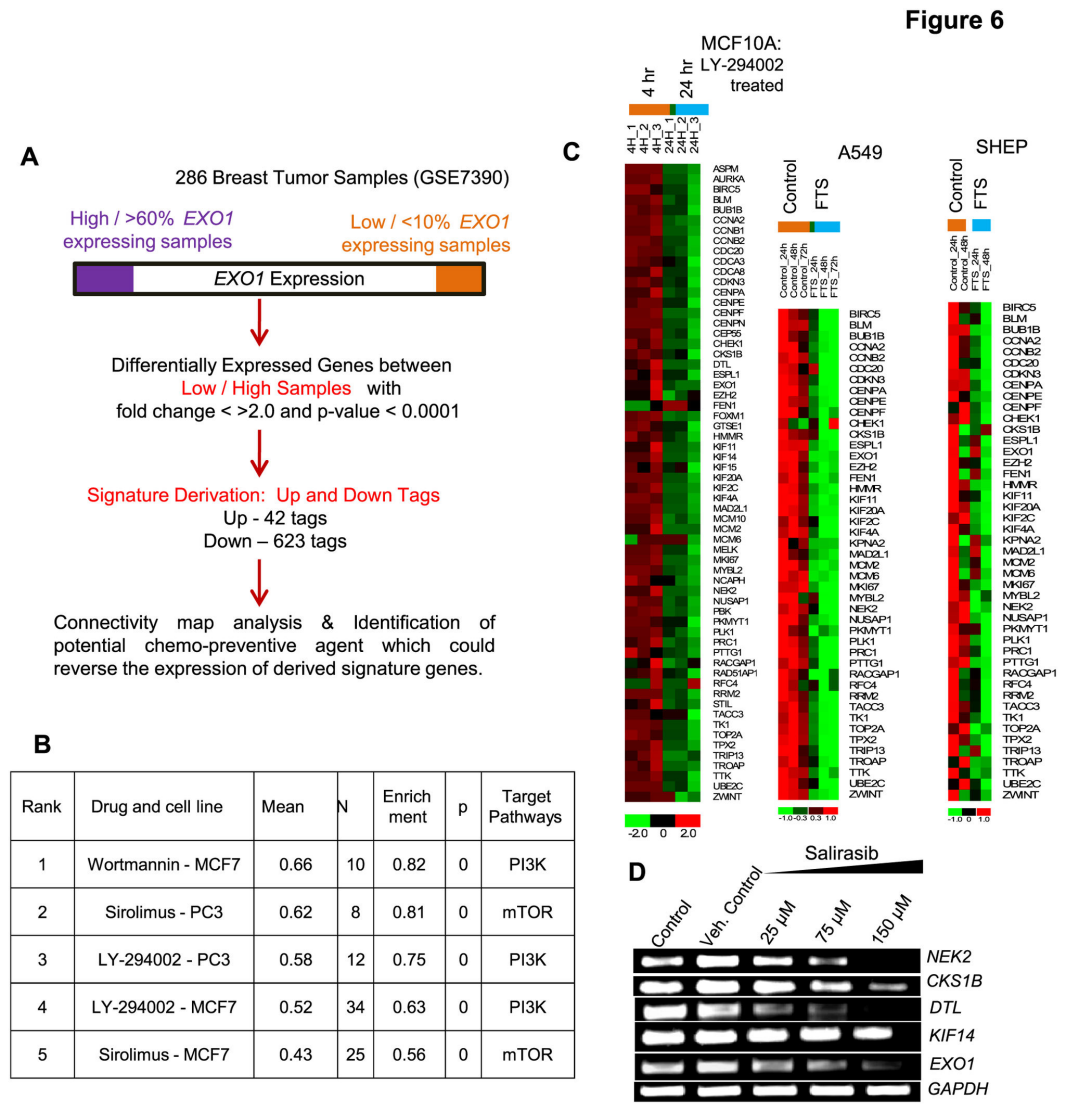
Ras/PI3K inhibition is the suitable therapeutic approach for breast tumors with elevated *EXO1* modular expression. (A) Workflow employed for the identification of suitable chemo therapeutic agent for *EXO1* module expressing breast tumors. (B) Results of Connectivity map analysis showing that while patients expressing *EXO1* co-regulated genes, Pi3K inhibition could be the possible targeted therapeutic approach. (C) Heatmap showing the inhibition of *EXO1* modular genes in GSE33403 downloaded from GEO that corresponds to MCF10A cells (PIK3CA E545K mutation) treated with LY294002 for 4 hours and 24 hours. Similarly, the profiles of A549 and SHEP cells treated with the Ras inhibitor FTS were obtained from Blum et al., 2007. (D) RT-PCR showed reduced expression of 5 out of 7, 1q candidate genes *NEK2, CKS1B, DTL, KIF14* and *EXO1* in MB231 cells upon chemical inhibition of RAS pathway with salirasib.

With the identified clinically significant molecular pathological and disease stratification features of *EXO1* expression in breast cancer, the identification of suitable therapeutic strategy for targeting the breast cancer cells with elevated *EXO1* expression would be useful for the development of novel breast cancer therapeutic options. In breast tumor mRNA profiles (GSE7390), the genes differentially expressed between elevated *EXO1* expressing breast tumors (>60%) and reduced *EXO1* expressing breast tumors (<10%) were short-listed. The derived genes were analyzed in connectivity map [38] and obtained a list of drugs that could inhibit the expression of genes which are expressed in breast cancer patients with elevated *EXO1* expression (Figure 6A). This analysis revealed many PI3K inhibitors like sirolimus, LY-294002, and wortmanin to be potent inhibitors for the potential reversal of the expression of *EXO1* associated gene-set (Figure 6B). This is also in agreement with the delineated regulatory mode of *EXO1* which involves Ras/PI3K/AKT. In order to further assess this possibility, we analysed the expression of *EXO1* modular genes in the gene expression profiles of PI3K / RAS inhibitor treated cells which were readily available in microarray repository. This analysis in a mammary epithelial cell (MCF10A) and few non-breast originated cells (A549 and SHEP), upon treatment with the PI3K inhibitor (LY294002) and Ras inhibitor (FTS), showed tremendous inhibition of *EXO1* modular genes (Figure 6C). Similarly, inhibition of Ras by Salirasib also resulted in reduced expression of *EXO1* modular and 1q candidate genes *NEK2, CKS1B, DTL* and *KIF14* in MDA-MB-231 cells (Figure 6D). On the other hand, inhibition of PI3K activity in MDA-MB-468 cell lines with LY-294002 also showed decreasing *EXO1* expression in a dose dependent manner. All these support Ras/PI3K inhibition as a possible therapeutic strategy for *EXO1* modular activated breast tumors. 

## Discussion

Genomic abnormalities remain a characteristic feature of cancer cells. Despite being the frequent chromosomal aberration in breast cancer genomes, the candidate target genes of 1q remain unexplored. Understanding of the pathological roles of target genes may further lead to the development of novel breast cancer stratification and targeted therapeutics and would have implications in the effective management of breast cancer patients with 1q amplification. Through a meta-analysis work-flow, by analysing the clinical outcome of breast cancer patients in association with the expression of the genes individually, we scored the candidacy of all genes from 1q. Genome-wide mRNA profiles and survival data available for 6 different breast tumor cohorts were used for this analysis and we identified 7 candidate genes from chromosome 1q region. Earlier, these genes were independently noticed for their significant involvement in breast cancers. For instance, *CENPF* (Centrosome protein F) and *NEK2* (NIMA (never in mitosis gene a) - related kinase 2) were reported for their association with poor prognosis and chromosomal instability in breast cancer [27,29]. In our current filtering of 1q genes related to survival, *CENPF* (HR = 1.616; p-value = 4.90E-05) ranked first followed by *NEK2* (HR = 1.641; p-value = 0.000163) which demonstrates the reliability of the strategy and the results obtained in the study. Commonality with most of the short-listed genes (*CENPF, KIF14, NEK2, CKS1B* and *ASPM*) is their positive association with proliferation marker Ki-67 [26,27,30,45,46], thereby, indicating their possible role in cell cycle related dysregulations and the resultant proliferation of breast cancer cells. However, it has been reported that simultaneous 1q gain/16q loss was related to low Ki-67 level (low proliferation) and high p27 expression of breast cancer cells [17]. While simultaneously considering 1q gain and 16q loss, low proliferation rate which otherwise the consequence of 16q loss also might have masked the significance of 1q gain. Our results show clear association between 1q candidate gene expression and proliferation.

The candidate gene which is relatively underexplored in breast cancers is Exonuclease I (*EXO1*), a Rad2 family member possessing 5’-3’ exonuclease activity and well established for its role in mismatch repair and DNA recombination. Being a DNA mismatch repair gene which is known to play a role in maintaining genomic integrity, its strong candidature with poor clinical outcome among breast cancer patients need further investigation. *EXO1* mutant mice were reported to have reduced survival and increased susceptibility to lymphoma development [37]. Studies evaluating single nucleotide polymorphisms in DNA repair related genes have emphasized the role of *EXO1*-K589E allele as a biomarker potentially linked with carcinogenesis [34-36]. A recent study showed the elevated expression of *EXO1* in ductal carcinoma in situ samples and is the first hint wherein the role of *EXO1* in breast cancer was revealed [33]. In the current integrative genomic investigation, analysis of *EXO1* gene expression across various groups and subtypes of breast cancer reveal *EXO1*’s higher expression in higher grade, basal and ER negative subtypes. In the same manner, a pattern of elevated expression in ER negative breast cancer was reported for 2 of the 1q candidate genes *CENPF* [27] and *KIF14* [28]. *NEK2* was reported for marked expression with both ER positive and negative subtypes [29]. Our analysis across breast cancer and non-cancerous breast mRNA profiles showed all 7 candidate genes to have similar expression pattern, apart from their association with poor clinical outcome. Since this pattern identification is from 4 different cohorts of breast tumors from 1371 breast cancer tissues, this would be more reliable than the previously mentioned single cohort based studies. 

**Figure 7:**
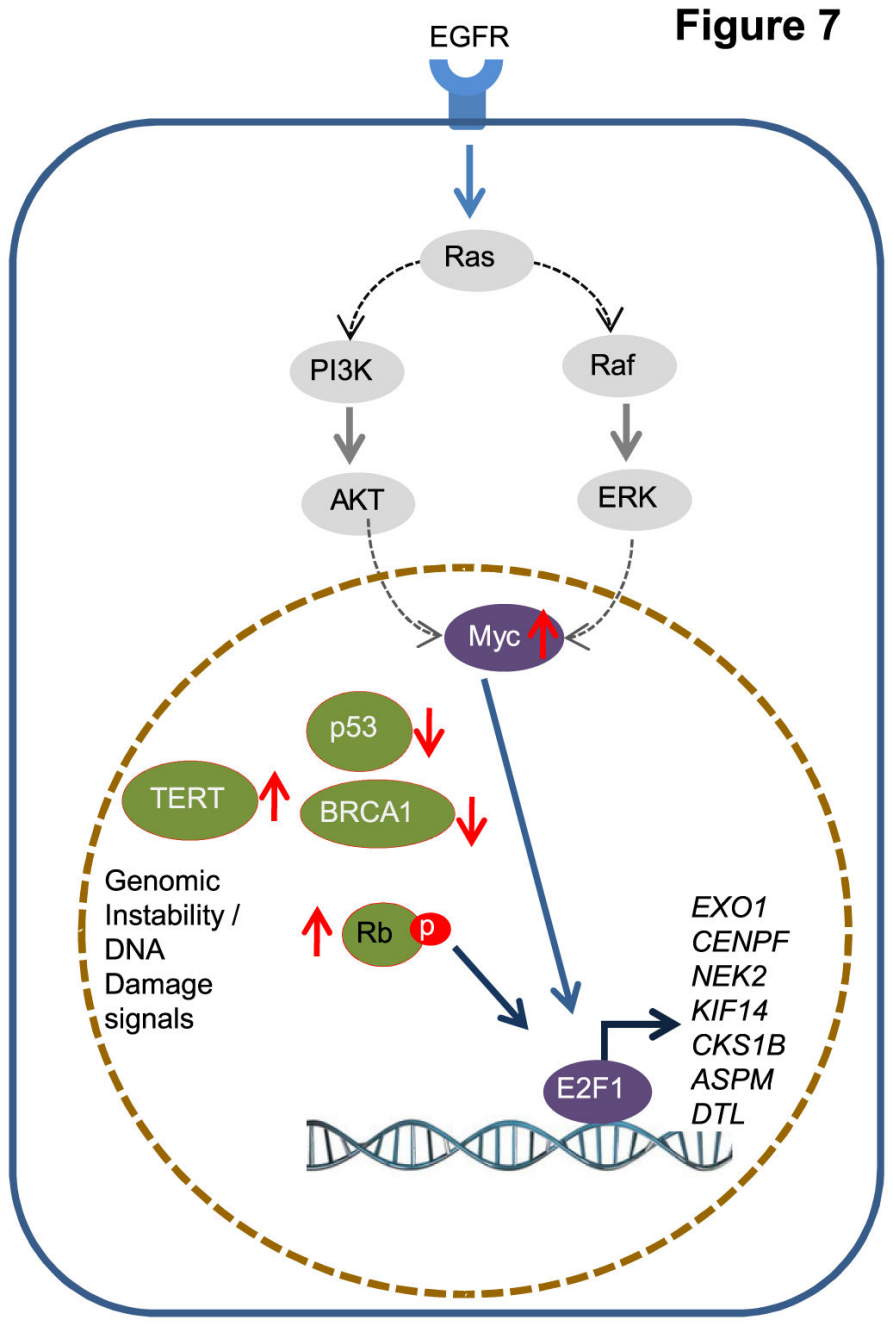
Schematic representation of signaling pathways/factors possibly regulating 1q candidate gene expression in breast cancer. EGFR, RAS, PI3K / AKT, MYC, and E2F were identified as the upstream regulators of these genes. The sequential arrangement of pathways is from the well established literature [49-51]. Apart from the pathways, genomic instability, telomerase activation and loss of p53 are also associated with the expression of 1q candidate genes.

Due to 1q amplification and very similar expression pattern, we hypothesized all 7 genes to have a similar regulatory pattern and investigated *EXO1* as a representative candidate from 1q region. Unravelling the factors regulating *EXO1* by signaling pathway focused gene set activation pattern prediction revealed closer association of activated RAS, EGFR, MYC and E2F pathways with *EXO1*’s elevated expression. In addition, genomic instability and telomerase activations showed positive association with *EXO1* gene expression. This association is prominent in breast tumors and breast cancer cells. RAS oncogenes (KRAS, HRAS and NRAS) harbour activation mutation in about 20% of human tumors and confers uncontrolled cell proliferation advantage [47]. In breast cancers, RAS is often activated by HER2 [ErbB2/epidermal growth factor receptor (EGFR) 2/Neu] receptor tyrosine kinase and is over expressed and persistently activated in approximately 25 % of cancers [48]. We observed a striking concordance between the expression pattern of *EXO1* (Figure 3D) and RAS activation pattern in breast cancer cell lines reported earlier [47]. This strongly suggests the intimate positive association between *EXO1* expression and RAS activation. Apart from RAS being a possible regulator of *EXO1*, the striking observation is, ‘the expression of *EXO1* is indicative of RAS activation’.The effector signals downstream of RAS could be ERK/MAPK signalling or PI3K/AKT signalling which are involved in cell survival and proliferation [49]. PI3K/AKT signalling is also reported to stabilize c-MYC expression in GSK3B dependent manner and the transcription of E2F1 gene thereby leading to S-phase progression in cell cycle [50]. Ras also regulates the expression of E2F [51]. First, in the light of these literature evidences, second, with closer association of activated RAS, EGFR, MYC and E2F signaling with *EXO1* expression, and third with the series of *in vitro* experiments (Figure 3 & 4), for the first time we report the regulation of *EXO1* in breast cancer cells through EGFR/RAS/PI3K/AKT/MYC/E2F signaling cascades (Figure 7). However, the direct upstream regulator of *EXO1* needs to be determined. Since *EXO1* is identified from analysing a frequently aberrated genomic region, its association with genomic instability and loss of p53 activity is not surprising and indeed supports the delineated regulatory modes of *EXO1*. Earlier report shows that frequent loss of PTEN could contribute to genomic instability in triple negative breast tumors [52]. This also strengthens the involvement of RAS/PI3K/MYC/E2F pathways in conferring genomic instability, and *EXO1* modular expression is indicative of that.

We also assessed the involvement of *EXO1* in DNA repair in breast cancer cells. Exposure of breast cancer cells to alkylating and DNA damaging agents induces the expression of *EXO1*. In view of the observed correlation between elevated *EXO1* expression and activated genomic instability related gene-set in breast tumors (Figure 3A), this observation suggests the possibility that prolonged *EXO1* expression in breast cancer is indicative of un-rectified DNA repairs. This shows that apart from being a poor survival indicator, *EXO1* expression in breast cancers is also indicative of cancers with elevated genomic instability. However, the functional role of *EXO1* in DNA repair needs to be investigated further. Our pathway focused analysis also supports the observation of overall elevated genomic instability in ER negative, basal and aggressive breast tumors [52,53]. Further, activated RAS/PI3K/MYC/E2F signaling is also the feature of these ER negative, basal and aggressive breast tumors. Importantly, *EXO1* expression is indicative for all these features in breast cancers. 

Using the concept of co-expression, we identified the common thread connecting *EXO1* gene with other genes in breast cancer (*EXO1* module) to be cell cycle progression and proliferation (Figure 5A). Cellular proliferation stands the traditional marker for prognosis that has different predictive values in ER positive and ER negative cancers [6], and works far beyond ER status [54]. Strikingly, in the derived *EXO1* modular gene-set, all seven 1q candidate genes were observed. This also implies that the delineated regulation of *EXO1* is applicable to all *EXO1* modular genes and in particular to all the seven 1q genes identified in this investigation. Supporting this notion, 44 out of 63 *EXO1* module genes were earlier identified to be regulated by RAS [55]. This also illustrates the possible 1q amplification independent regulation of *EXO1* module in breast cancers. Since only 7 out of the 498 genes located in 1q amplicon are selectively expressed in breast cancers, 1q amplification independent and specifically regulated *EXO1* modular expression is quite possible in breast cancers and needs to be investigated. 

*EXO1* module represents a highly conserved set of interactions in breast cancer. Comparing *EXO1* module with different sets of gene signatures revealed very less overlap with clinically used gene signatures like MammaPrint and Oncotype and other breast cancer derived prognostic gene expression signatures (Figure S7). The observed minimal overlaps among the other signatures were pointing the proliferation related genes as common denominator for several prognostic gene expression signatures analysed in this study. Wound response and CIN70 gene signatures, which were earlier demonstrated as powerful predictors of metastasis in multiple cancers [56,57] showed higher overlap with *EXO1* module in terms of gene content (Table S8). Accordingly, *EXO1* module genes’ expression is also observed to be higher in metastatic and basal breast tumors. *EXO1* module genes are comprised of cellular proliferation related genes and thus represent i) cellular proliferation, ii) activated RAS/PI3K/MYC/E2F signaling, iii) elevated genomic instability and loss of p53 functionality, and iv) better predictor of metastasis and poor clinical outcome. 

Elevated expression of *EXO1* in ER negative and high grade breast tumors indicate the possible development of targeted therapeutics by targeting *EXO1* module or its upstream regulators. Connecting the gene expression with possible chemical inhibition through the concept of connectivity map showed that RAS/PI3K inhibitors could inhibit the expression of *EXO1* modular genes. With evidences from the connectivity map and *in-vitro* gene expression analysis upon treatment with PI3K or RAS inhibitor, it seems possible to target PI3K or RAS in order to inhibit *EXO1* modular expression in breast cancer cells. While considering the identified upstream regulators of *EXO1*, targeting EGFR, RAS or PI3K is also possible. A number of clinical trials targeting EGFR, PI3K and RAS downstream signals were performed recently. Anti-EGFR therapies yielded Gefitinib and Erlotinib, anti-PI3K therapies yielded Rapamycin and Tipifarnib that inhibits farnesylation of RAS and other proteins involved in signal transduction pathways [47,58,59]. Despite being the activator of RAS signalling pathway, EGFR derives less attention in this aspect due to activating mutations in KRAS, which are significantly associated with lack of response or resistance with some of the EGFR inhibitors like cetuximab in colorectal and lung cancers [58,60]. Therefore, as per the predictions and *in vitro* validation, RAS, or PI3K inhibition could be considered for evaluating the potential inclusion in the regimen for *EXO1* over expressing group of patients. However, in prior, the KRAS and PIK3CA mutations status also needs to be considered. It is worth mentioning that *EXO1* over expressing group of patients also could be denoted as 1q candidate gene over expressing group of patients. 

## Conclusion

In this study, we scanned chromosome 1q genes for their significant association with survival of the patients and identified 7 potential candidate genes. These genes were found to consistently over express in high grade and aggressive breast tumors with poor clinical outcome. We delineated the upstream regulators of *EXO1*, an underexplored candidate gene in breast cancer. By integrative functional genomics and molecular cell biological approaches, we showed the involvement of EGFR, RAS, PI3K / AKT, MYC, E2F signaling in the regulation of these selected 1q genes in breast tumors. Expression of *EXO1* module, the gene set derived from co-expressed genes of *EXO1* gene, was found as indicative of elevated cell proliferation, genomic instability, activated RAS/AKT/MYC/E2F1 signaling pathways and loss of p53 activity in breast tumors. We also suggest the inhibition of RAS/PI3K as possible therapeutic option for the patients with elevated expression of the *EXO1* module.

## Supporting Information

Figure S1 **Investigation of *EXO1* gene expression in different categories of breast tumors.** Expression pattern of EXO1 in (A) breast tumors while compared to normal breast tissues. (B) PR positive and negative tumors, (C) metastatic tumors against non-metastatic groups, (D) Invasive Ductal Carcinoma (IDC), Invasive Lobular Carcinoma (ILC) and mixed sub-types.
(TIF)

Figure S2 **EXO1 expression is elevated in basal type of breast tumors.** Expression pattern of *EXO1* across basal, luminal and HER2 subtypes of breast cancer in 3 different datasets (A-C).
(TIF)

Figure S3 **Pathway activation pattern in breast cancer cell lines.** (A) Heatmap showing pathway activation pattern in breast cancer cell lines (E-TABM-157). (B) Principal component analysis of pathway activation scores in breast cancer cell lines.
(TIF)

Figure S4 **Gene expression pattern of EXO1 in a panel of breast cancer cell lines.** *EXO1* expression in breast cancer cell lines as extracted from E-TABM-157 profile was showed as bar chart.
(TIF)

Figure S5 **NFY and E2F transcription factors are enriched in EXO1 module.** Analysis on transcription factor binding sites enrichment in *EXO1* module revealed the higher percentage of NFY and E2F transcription factors.
(TIF)

Figure S6 **EXO1 module genes show higher expression in high grade, aggressive breast tumors.** Expression pattern of *EXO1* module in two different breast tumor profiles (A) GSE25066 and (B) GSE7390 is depicted as heatmap.
(TIF)

Figure S7 **Comparison of the overlap between *EXO1* modular genes and other prognostic gene sets of breast tumors.**
(TIF)

Table S1 **List of datasets used in the study.**
(DOCX)

Table S2 **Results of Meta-analysis on breast cancer survival data for 1q genes.**
(XLSX)

Table S3 **Univariate and Multivariate analysis with *EXO1* gene expression and other clinical covariates reveals the prognostic significance of *EXO1* in breast cancer.**
(DOCX)

Table S4 **List of pathway signatures used for predicting upstream regulators of *EXO1*.**
(DOCX)

Table S5 **Regression analysis of *EXO1* gene expression with pathway activation status in 198 breast tumor samples (GSE7390).**
(DOCX)

Table S6 **Regression analysis of *EXO1* gene expression with pathway activation status in 51 breast cancer cell lines (E-TABM-157).**
(DOCX)

Table S7 **Results of Meta-analysis of correlation coefficients.**
(XLSX)

Table S8 **List of breast cancer signatures used for *EXO1* module comparison.**
(DOCX)
