# Supplementary figures and images for: Breast Tumors with Elevated Expression of 1q Candidate Genes Confer Poor Clinical Outcome and Sensitivity to Ras/PI3K Inhibition
Source: PLoS One. 2013 Oct 17;8(10):e77553. doi: 10.1371/journal.pone.0077553 (PMC3798322; doi:10.1371/journal.pone.0077553)

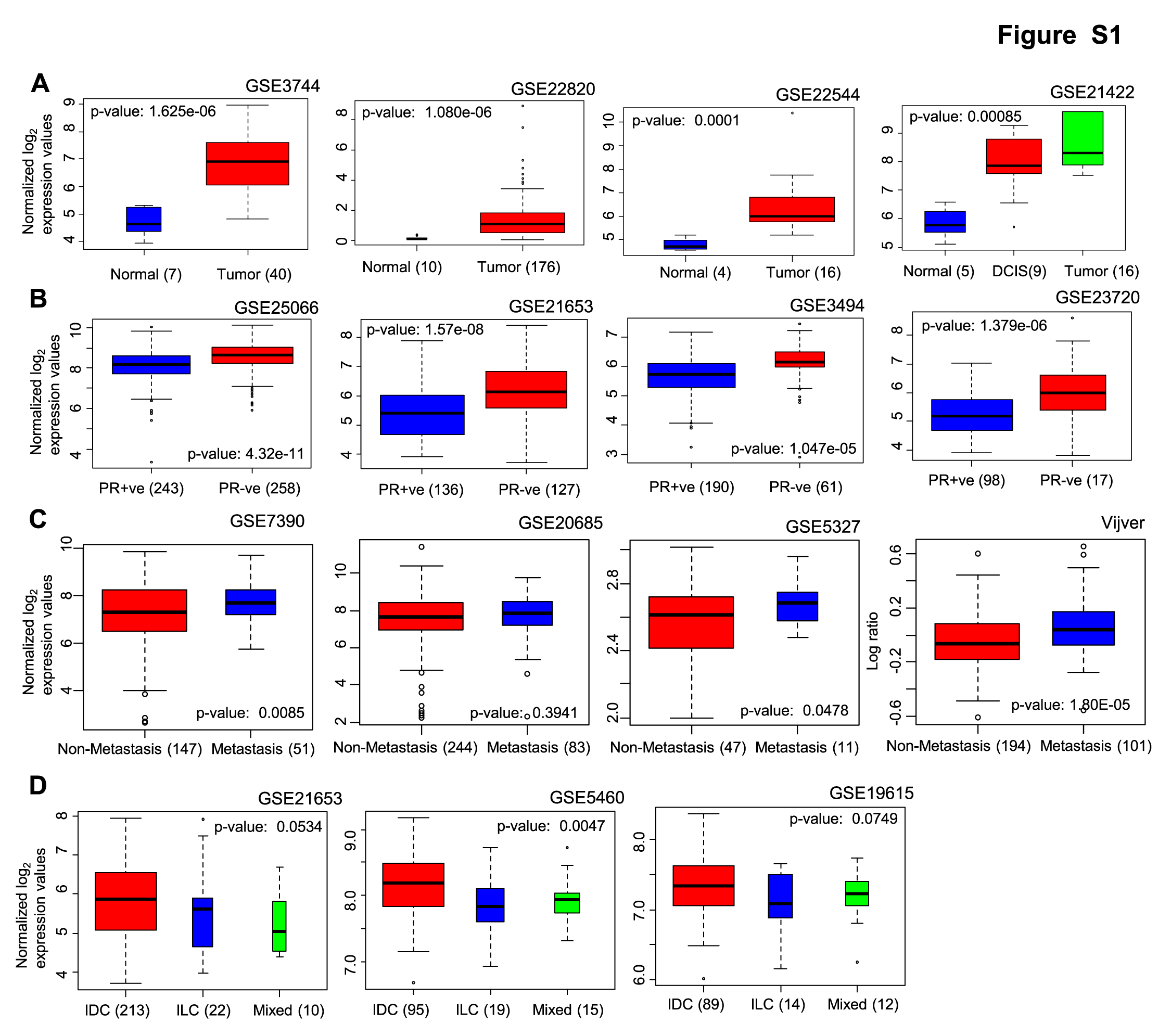

Supplement: Figure S1 — Investigation of EXO1 gene expression in different categories of breast tumors. Expression pattern of EXO1 in (A) breast tumors while compared to normal breast tissues. (B) PR positive and negative tumors, (C) metastatic tumors against non-metastatic groups, (D) Invasive Ductal Carcinoma (IDC), Invasive Lobular Carcinoma (ILC) and mixed sub-types. (TIF) [file pone.0077553.s001.tif]

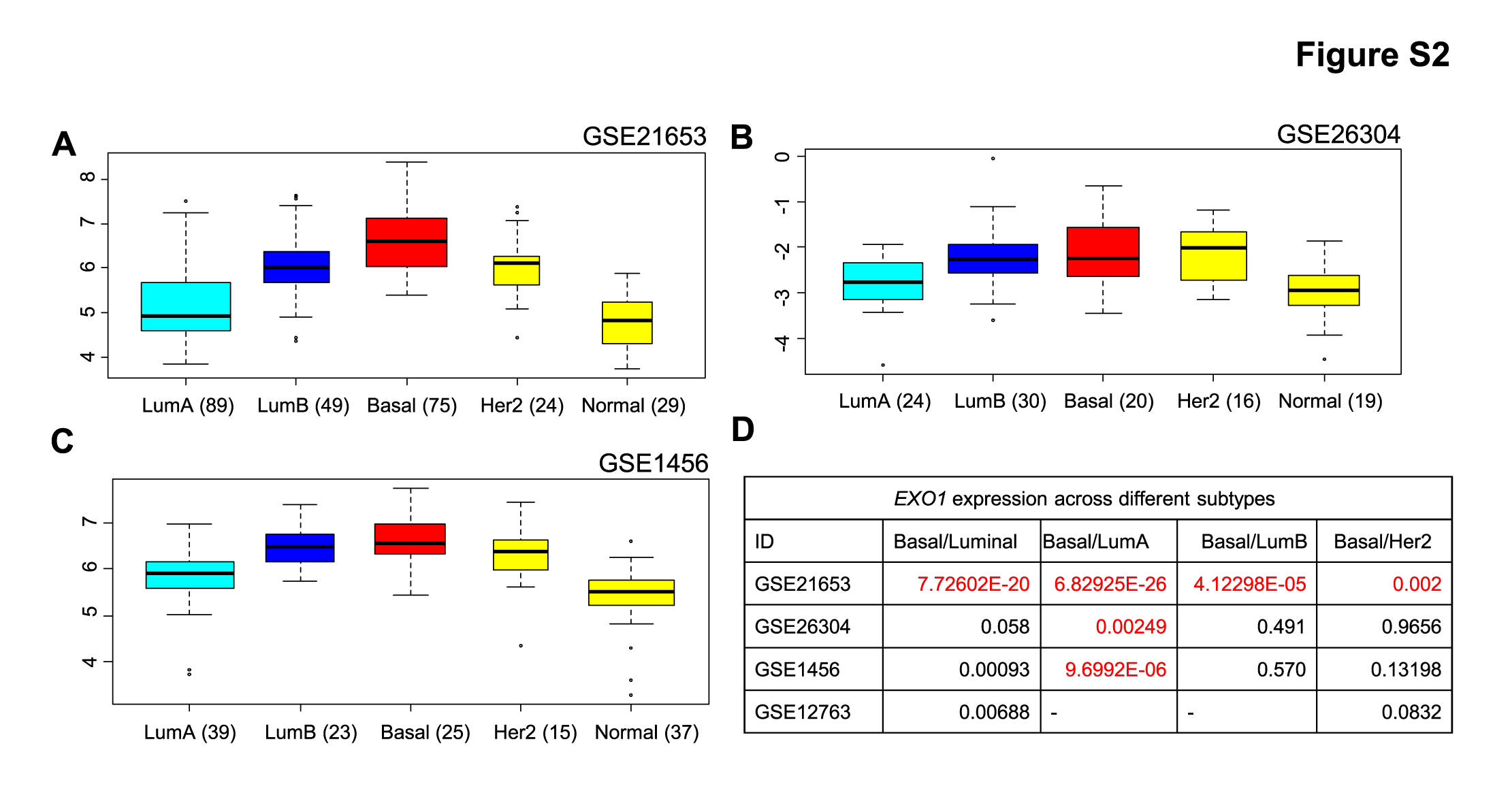

Supplement: Figure S2 — EXO1 expression is elevated in basal type of breast tumors. Expression pattern of EXO1 across basal, luminal and HER2 subtypes of breast cancer in 3 different datasets (A-C). (TIF) [file pone.0077553.s002.tif]

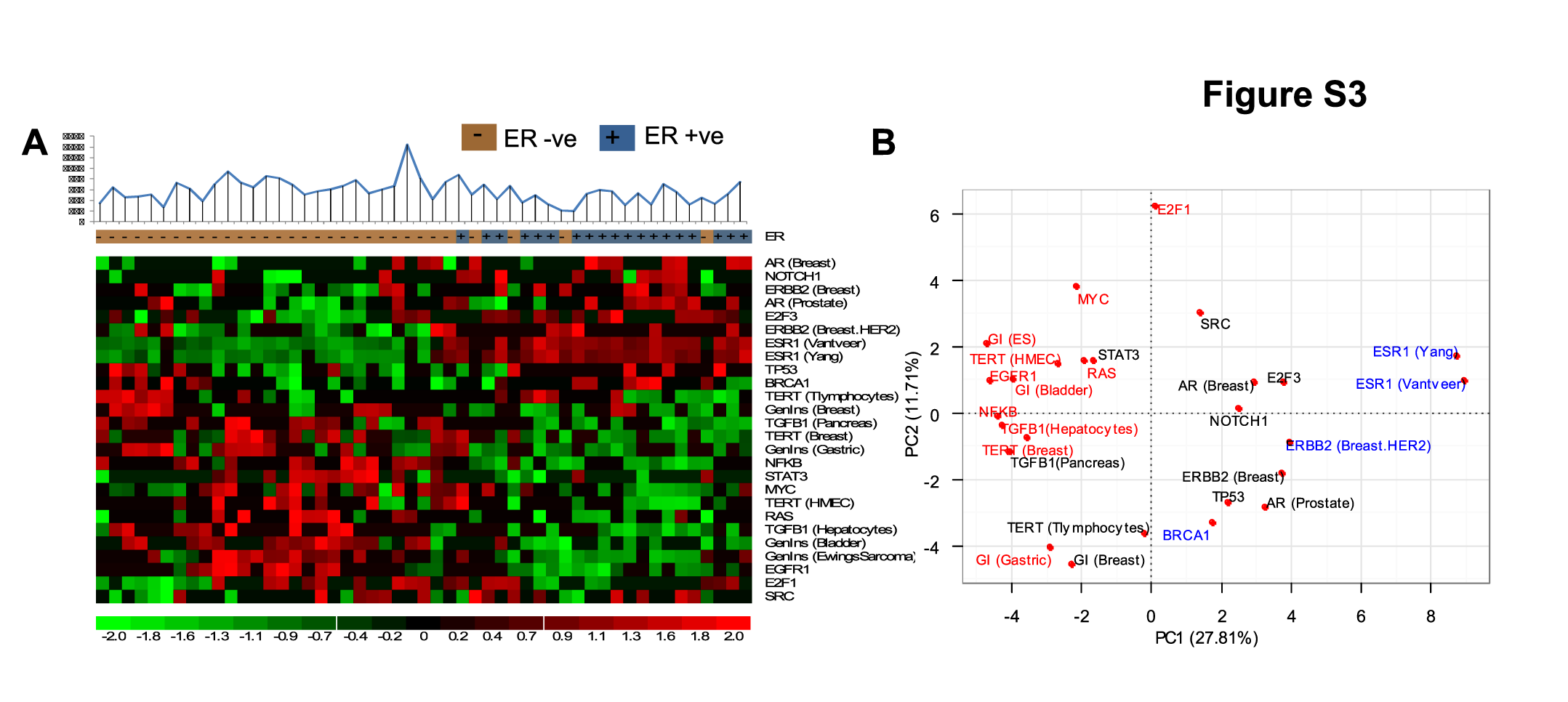

Supplement: Figure S3 — Pathway activation pattern in breast cancer cell lines. (A) Heatmap showing pathway activation pattern in breast cancer cell lines (E-TABM-157). (B) Principal component analysis of pathway activation scores in breast cancer cell lines. (TIF) [file pone.0077553.s003.tif]

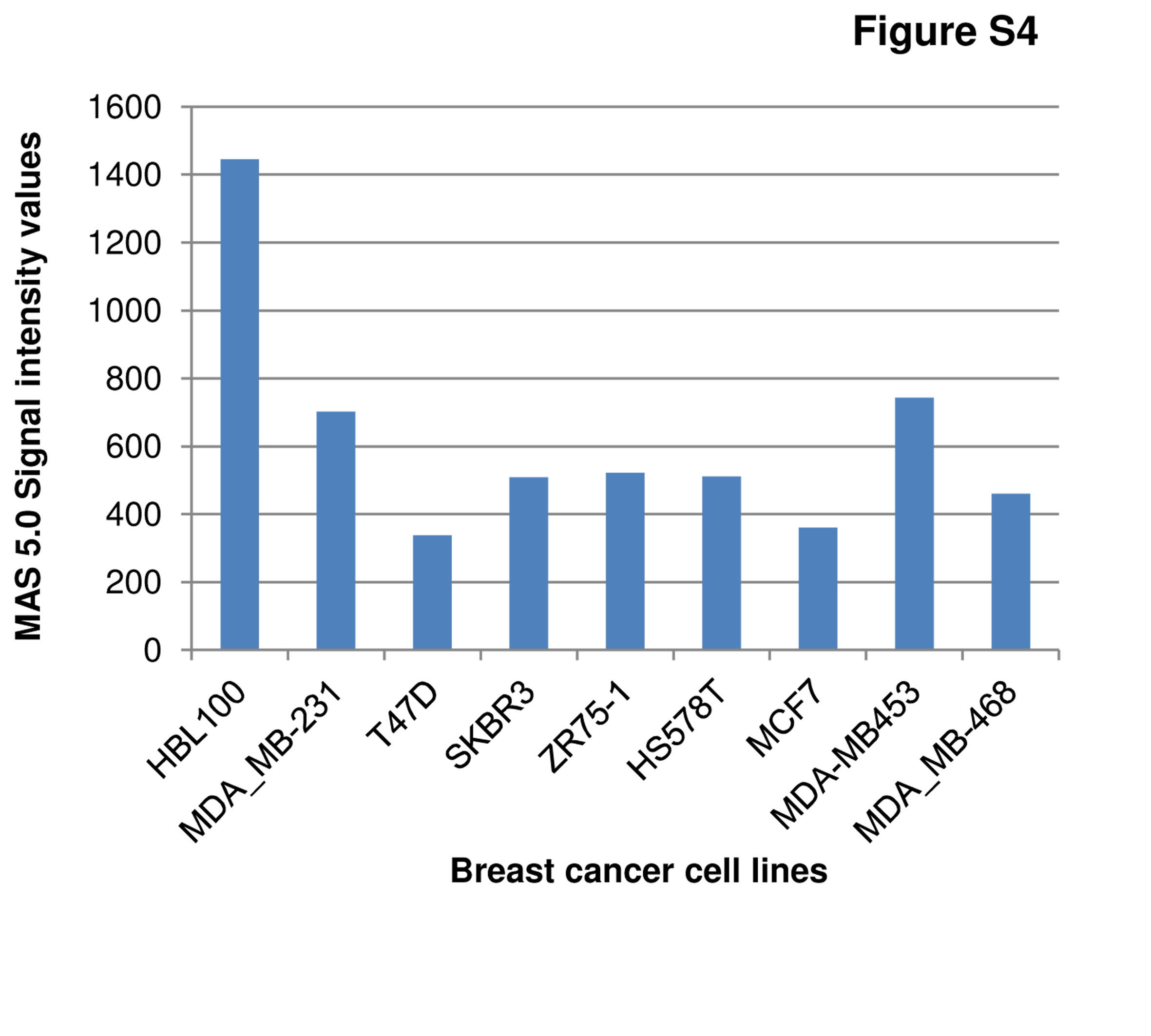

Supplement: Figure S4 — Gene expression pattern of EXO1 in a panel of breast cancer cell lines. EXO1 expression in breast cancer cell lines as extracted from E-TABM-157 profile was showed as bar chart. (TIF) [file pone.0077553.s004.tif]

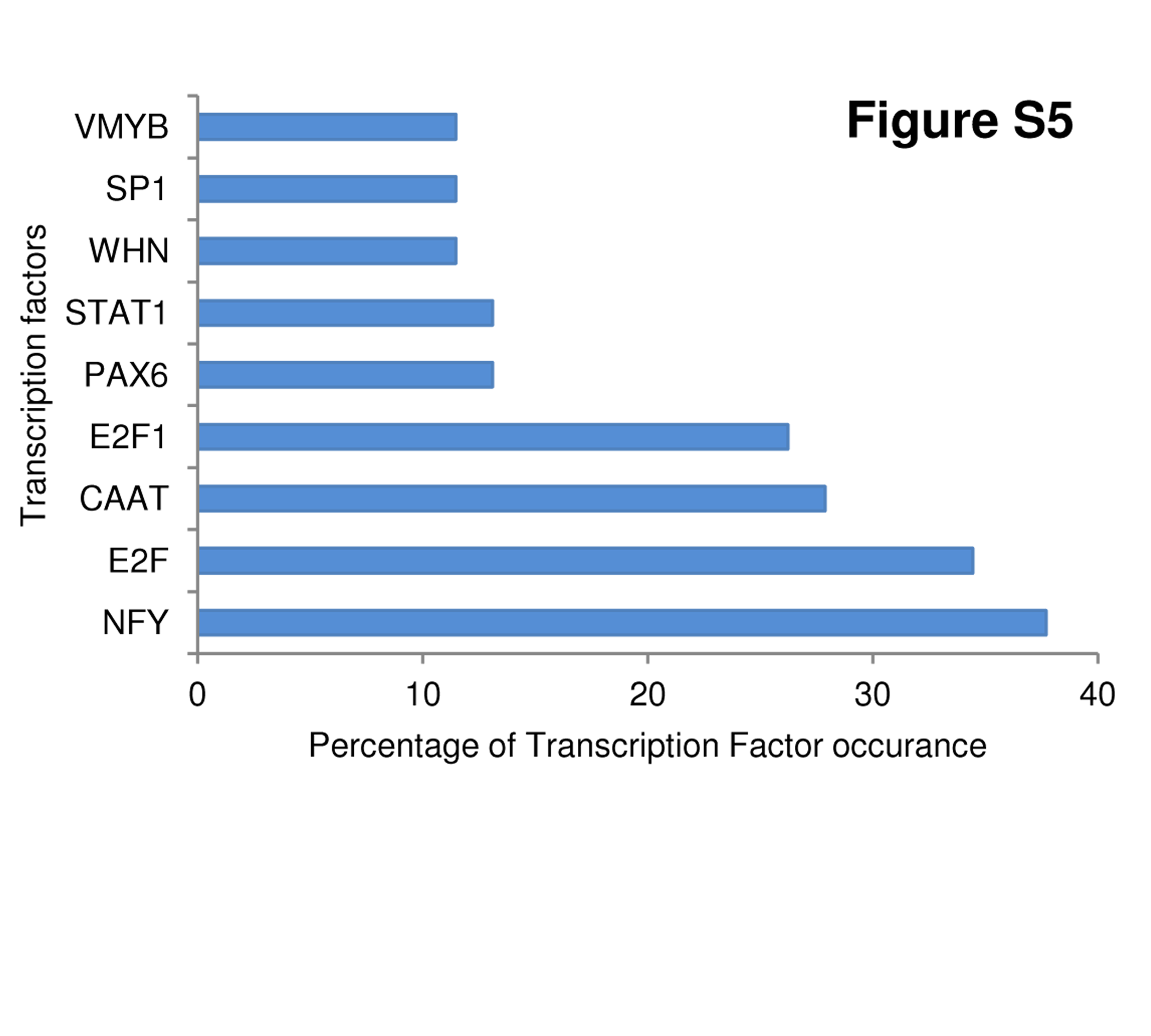

Supplement: Figure S5 — NFY and E2F transcription factors are enriched in EXO1 module. Analysis on transcription factor binding sites enrichment in EXO1 module revealed the higher percentage of NFY and E2F transcription factors. (TIF) [file pone.0077553.s005.tif]

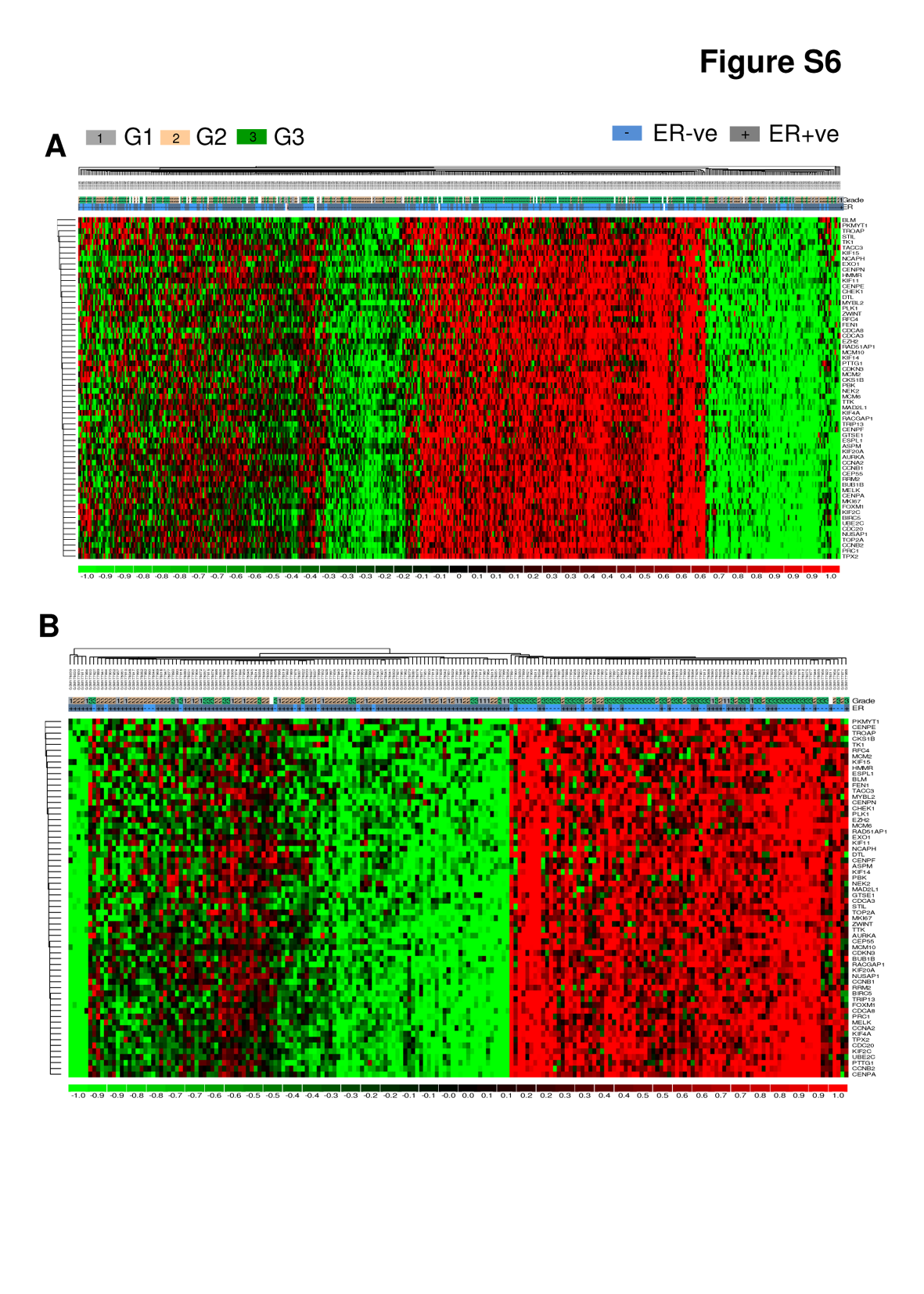

Supplement: Figure S6 — EXO1 module genes show higher expression in high grade, aggressive breast tumors. Expression pattern of EXO1 module in two different breast tumor profiles (A) GSE25066 and (B) GSE7390 is depicted as heatmap. (TIF) [file pone.0077553.s006.tif]

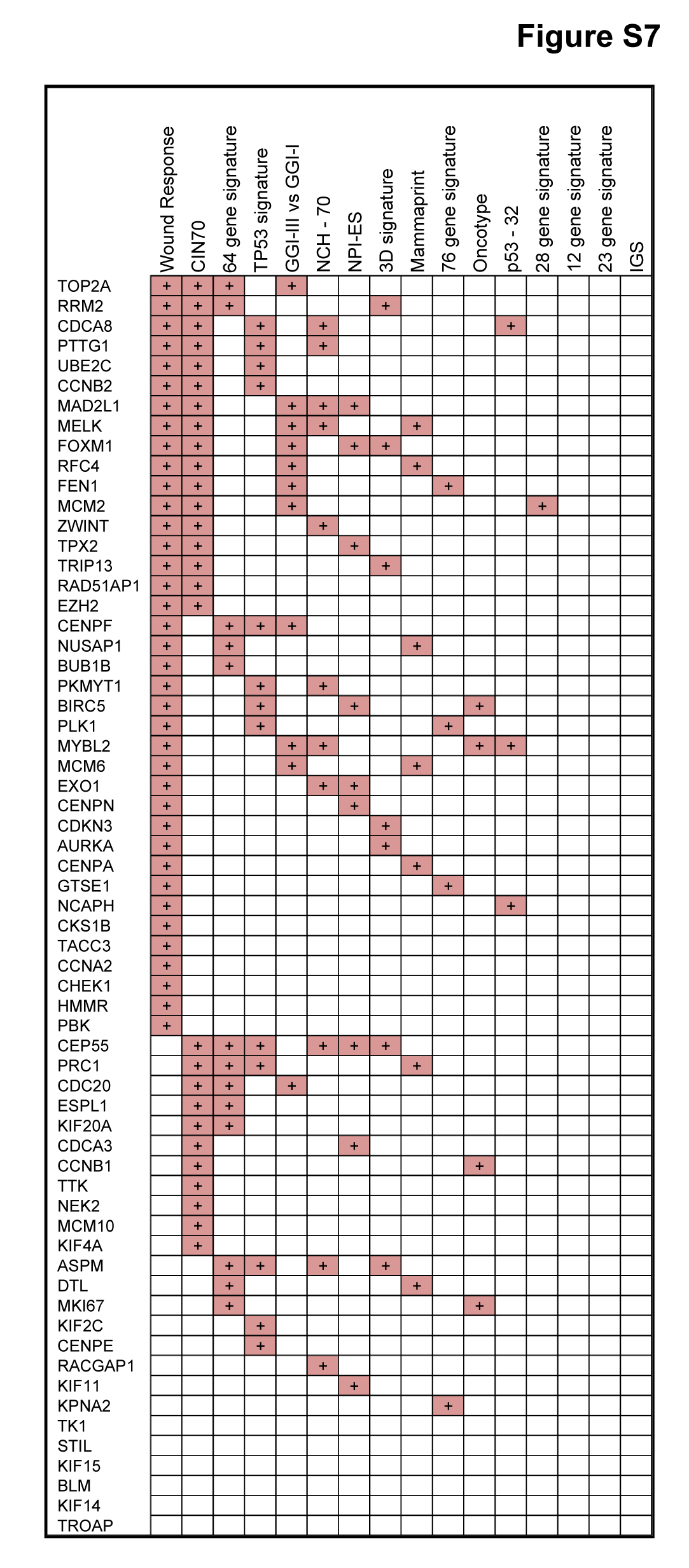

Supplement: Figure S7 — Comparison of the overlap between EXO1 modular genes and other prognostic gene sets of breast tumors. (TIF) [file pone.0077553.s007.tif]
