# Supplementary material for: Breast Tumors with Elevated Expression of 1q Candidate Genes Confer Poor Clinical Outcome and Sensitivity to Ras/PI3K Inhibition
Source: PLoS One. 2013 Oct 17;8(10):e77553. doi: 10.1371/journal.pone.0077553 (PMC3798322; doi:10.1371/journal.pone.0077553)
Supplement: Table S1 — List of datasets used in the study. (DOCX) [file pone.0077553.s008.docx]

**Table S1.** List of datasets used in the study.

| **GEO Accession ID** | **Reference** | **Total number of samples (Normal /Tumor)** | **Normal/Tumor** | **Relapse Free Survival** | **Meta analysis of Correlation Coefficients** | **ER+ve/ER-ve** | **Grade** | **PR+ve/PR-ve** | **Metastasis** | **Histological subgroups** | **Subtypes (Basal/LuminalA/LuminalB/ Normal** |
| --- | --- | --- | --- | --- | --- | --- | --- | --- | --- | --- | --- |
| GSE25066 | [[1](#_ENREF_1)] | 508 |  | y | y | y | y | y |  |  |  |
| GSE3494 | [[2](#_ENREF_2)] | 251 |  |  | y |  | y | y |  |  |  |
| GSE7390 | [[3](#_ENREF_3)] | 198 |  | y | y | y | y |  | y |  |  |
| Vijver | [[4](#_ENREF_4)] | 295 |  | y | y | y | y |  | y |  |  |
| GSE2034 | [[5](#_ENREF_5)] | 286 |  | y | y |  |  |  |  |  |  |
| GSE21653 | [[6](#_ENREF_6)] | 266 |  |  | y |  |  | y |  | y | y |
| GSE20685 | [[7](#_ENREF_7)] | 327 |  |  | y |  |  |  | y |  |  |
| GSE1456 | [[8](#_ENREF_8)] | 159 |  | y | y |  |  |  |  |  | y |
| GSE2990 | [[9](#_ENREF_9)] | 189 |  | y | y |  |  |  |  |  |  |
| GSE23720 | [[10](#_ENREF_10)] | 370 |  |  |  | y |  | y |  |  |  |
| GSE5460 | [[11](#_ENREF_11)] | 129 |  |  |  |  |  |  |  | y |  |
| GSE19615 | [[12](#_ENREF_12)] | 115 |  |  |  |  |  |  |  | y |  |
| GSE5327 | [[13](#_ENREF_13)] | 58 |  |  |  |  |  |  | y |  |  |
| GSE26304 | - | 115 |  |  |  |  |  |  |  |  | y |
| GSE3744 | [[14](#_ENREF_14)] | 47(7/40) | y |  |  |  |  |  |  |  |  |
| GSE22820 | [[15](#_ENREF_15)] | 186(10/176) | y |  |  |  |  |  |  |  |  |
| GSE22544 | [[16](#_ENREF_16)] | 20 (4/16) | y |  |  |  |  |  |  |  |  |
| GSE21422 | [[17](#_ENREF_17)] | 30 (5/25) | y |  |  |  |  |  |  |  |  |
| Total no. of samples used |  | 26/3523 | 26/257 | 1635 | 2479 | 1371 | 1252 | 1395 | 878 | 510 | 540 |

*symbol “y” denotes that the corresponding profile is used for the analysis

**References**

1. Hatzis C, Pusztai L, Valero V, Booser DJ, Esserman L, et al. (2011) A genomic predictor of response and survival following taxane-anthracycline chemotherapy for invasive breast cancer. JAMA 305: 1873-1881.

2. Miller LD, Smeds J, George J, Vega VB, Vergara L, et al. (2005) An expression signature for p53 status in human breast cancer predicts mutation status, transcriptional effects, and patient survival. ProcNatlAcadSciUSA %20;102: 13550-13555.

3. Desmedt C, Piette F, Loi S, Wang Y, Lallemand F, et al. (2007) Strong time dependence of the 76-gene prognostic signature for node-negative breast cancer patients in the TRANSBIG multicenter independent validation series. ClinCancer Res 13: 3207-3214.

4. van de Vijver MJ, He YD, van't Veer LJ, Dai H, Hart AA, et al. (2002) A gene-expression signature as a predictor of survival in breast cancer. NEnglJMed %19;347: 1999-2009.

5. Wang Y, Klijn JG, Zhang Y, Sieuwerts AM, Look MP, et al. (2005) Gene-expression profiles to predict distant metastasis of lymph-node-negative primary breast cancer. Lancet %19-25;365: 671-679.

6. Sabatier R, Finetti P, Cervera N, Lambaudie E, Esterni B, et al. (2011) A gene expression signature identifies two prognostic subgroups of basal breast cancer. Breast Cancer ResTreat 126: 407-420.

7. Kao KJ, Chang KM, Hsu HC, Huang AT (2011) Correlation of microarray-based breast cancer molecular subtypes and clinical outcomes: implications for treatment optimization. BMCCancer 11:143. doi: 10.1186/1471-2407-11-143.: 143-111.

8. Pawitan Y, Bjohle J, Amler L, Borg AL, Egyhazi S, et al. (2005) Gene expression profiling spares early breast cancer patients from adjuvant therapy: derived and validated in two population-based cohorts. Breast Cancer Res 7: R953-R964.

9. Sotiriou C, Wirapati P, Loi S, Harris A, Fox S, et al. (2006) Gene expression profiling in breast cancer: understanding the molecular basis of histologic grade to improve prognosis. JNatlCancer Inst 98: 262-272.

10. Richardson AL, Wang ZC, De NA, Lu X, Brown M, et al. (2006) X chromosomal abnormalities in basal-like human breast cancer. Cancer Cell 9: 121-132.

11. Liu RZ, Graham K, Glubrecht DD, Germain DR, Mackey JR, et al. (2011) Association of FABP5 expression with poor survival in triple-negative breast cancer: implication for retinoic acid therapy. AmJPathol 178: 997-1008.

12. Hawthorn L, Luce J, Stein L, Rothschild J (2010) Integration of transcript expression, copy number and LOH analysis of infiltrating ductal carcinoma of the breast. BMCCancer 10:460. doi: 10.1186/1471-2407-10-460.: 460-410.

13. Kretschmer C, Sterner-Kock A, Siedentopf F, Schoenegg W, Schlag PM, et al. (2011) Identification of early molecular markers for breast cancer. MolCancer 10: 15-10.

14. Lu X, Lu X, Wang ZC, Iglehart JD, Zhang X, et al. (2008) Predicting features of breast cancer with gene expression patterns. Breast Cancer ResTreat 108: 191-201.

15. Li Y, Zou L, Li Q, Haibe-Kains B, Tian R, et al. (2010) Amplification of LAPTM4B and YWHAZ contributes to chemotherapy resistance and recurrence of breast cancer. NatMed 16: 214-218.

16. Minn AJ, Gupta GP, Padua D, Bos P, Nguyen DX, et al. (2007) Lung metastasis genes couple breast tumor size and metastatic spread. ProcNatlAcadSciUSA 104: 6740-6745.

17. Chen DT, Nasir A, Culhane A, Venkataramu C, Fulp W, et al. (2010) Proliferative genes dominate malignancy-risk gene signature in histologically-normal breast tissue. Breast Cancer ResTreat 119: 335-346.
