# Supplementary material for: Breast Tumors with Elevated Expression of 1q Candidate Genes Confer Poor Clinical Outcome and Sensitivity to Ras/PI3K Inhibition
Source: PLoS One. 2013 Oct 17;8(10):e77553. doi: 10.1371/journal.pone.0077553 (PMC3798322; doi:10.1371/journal.pone.0077553)
Supplement: Table S3 — Univariate and Multivariate analysis with EXO1 gene expression and other clinical covariates reveals the prognostic significance of EXO1 in breast cancer. (DOCX) [file pone.0077553.s010.docx]

**Table S3:** Univariate and Multivariate analysis with *EXO1* gene expression and other clinical covariates reveals the prognostic significance of *EXO1* in breast cancer.

| Clinical variables | **GSE7390** | | **GSE2990** | | **Vijver** | |
| --- | --- | --- | --- | --- | --- | --- |
|  | Hazard Ratio | p-value | Hazard Ratio | p-value | Hazard Ratio | p-value |
| **Univariate Analysis** | | | | | | |
| *EXO1* | 1.58 [1.04 - 2.34] | 0.031 | 2.48 [1.50 - 4.09] | 0.000 | 2.30 [1.55 - 3.41] | 0.000 |
| ER | 1.31 [0.85 - 2.02] | 0.218 | 1.42 [0.83 - 2.46] | 0.204 | 1.96 [1.28 - 3.00] | 0.002 |
| Age | | | | | | |
| 40>=Age<=60 | 0.84 [0.50 - 1.39] | 0.491 | 0.34 [0.16 - 0.73] | 0.005 | 0.38 [0.25 - 0.58] | 0.000 |
| Age>60 |  |  | 0.55 [0.26 - 1.18] | 0.124 |  |  |
| Size  (mm) | | | | | | |
| 20>Size<=50 | 1.21 [0.80 - 1.83] | 0.356 | 1.97 [1.20 - 3.21] | 0.007 | 2.03 [1.36 - 3.03] | 0.001 |
| Size>50 |  |  | 5.65 [1.33 - 23.90] | 0.019 |  |  |
| Node | NA | NA | 0.96 [0.46 - 2.03] | 0.921 | 0.88 [0.60 - 1.30] | 0.520 |
| Grade | | | | | | |
| Grade 2 | 2.27 [1.07 - 4.81] | 0.032 | 2.18 [1.11 - 4.29] | 0.023 | 2.41 [1.22 - 4.76] | 0.012 |
| Grade 3 | 1.61 [0.75 - 3.51] | 0.221 | 1.74 [0.88 - 3.46] | 0.111 | 4.30 [2.26 - 8.21] | 0.000 |
| **Multivariate Analysis** | | | | | | |
| *EXO1* | 1.94 [1.62 - 3.23] | 0.011 | 2.08 [1.07 - 4.05] | 0.031 | 1.59 [1.00 - 2.54] | 0.049 |
| ER | 1.27 [0.74 - 2.19] | 0.381 | 0.93 [0.48 - 1.80] | 0.844 | 1.10 [0.68 - 1.80] | 0.690 |
| Age | | | | | | |
| 40>=Age<=60 | 0.96 [0.56 - 1.63] | 0.880 | 0.56 [0.24 - 1.31] | 0.184 | 0.44 [0.28 - 0.67] | 0.000 |
| Age>60 |  |  | 0.77 [0.33 - 1.80] | 0.543 |  |  |
| Size (mm) | | | | | | |
| 20>Size<=50 | 1.19 [0.77 - 1.84] | 0.422 | 1.99 [1.09 - 3.64] | 0.019 | 1.62 [1.07 - 2.47] | 0.023 |
| Size>50 |  |  | 6.95 [1.33 - 36.20] | 0.018 |  |  |
| Node | NA | NA | 0.61 [0.23 - 1.64] | 0.319 | 0.96 [0.63 - 1.45] | 0.836 |
| Grade | | | | | | |
| Grade 2 | 2.30 [1.08 - 4.90] | 0.031 | 1.59 [0.73 - 3.46] | 0.273 | 2.06 [1.03 - 4.10] | 0.041 |
| Grade 3 | 0.97 [0.41 - 2.29] | 0.940 | 0.88 [0.37 - 2.07] | 0.640 | 2.37 [1.15 - 4.86] | 0.020 |
