# Supplementary material for: Breast Tumors with Elevated Expression of 1q Candidate Genes Confer Poor Clinical Outcome and Sensitivity to Ras/PI3K Inhibition
Source: PLoS One. 2013 Oct 17;8(10):e77553. doi: 10.1371/journal.pone.0077553 (PMC3798322; doi:10.1371/journal.pone.0077553)
Supplement: Table S4 — List of pathway signatures used for predicting upstream regulators of EXO1. (DOCX) [file pone.0077553.s011.docx]

**Table S4.** List of pathway signatures used for predicting upstream regulators of *EXO1*

| **Signatures** | **Source** | **Key names** | **Description** |
| --- | --- | --- | --- |
| MYC | Bild AH, Yao G, Chang JT, Wang Q, Potti A, et al. (2006) Oncogenic pathway signatures in human cancers as a guide to targeted therapies. Nature 439: 353-357. | | |
| E2F3 |  |  |  |
| RAS |  |  |  |
| SRC |  |  |  |
| *ESR1*  (Vantveer) | MSigDb | VANTVEER_BREAST_CANCER_*ESR1* _UP | Up-regulated genes from the optimal set of 550 markers discriminating breast cancer samples by *ESR1*  [GeneID=2099] expression: ER(+) vs ER(-) tumors. |
|  |  | VANTVEER_BREAST_CANCER_*ESR1* _DN | Down-regulated genes from the optimal set of 550 markers discriminating breast cancer samples by *ESR1*  [GeneID=2099] expression: ER(+) vs ER(-) tumors. |
| STAT3 | MSigDb | DAUER_STAT3_TARGETS_UP | Top 50 genes up-regulated in A549 cells (lung cancer) expressing *STAT3* [Gene ID=6774] off an adenovirus vector. |
|  |  | DAUER_STAT3_TARGETS_DN | Top 50 genes down-regulated in A549 cells (lung cancer) expressing *STAT3* [Gene ID=6774] off an adenovirus vector. |
| E2F1 | Young AP, Nagarajan R, Longmore GD (2003) Mechanisms of transcriptional regulation by Rb-E2F segregate by biological pathway. Oncogene 22: 7209-7217. | | |
| *ESR1*  (Yang) | MSigDb | YANG_BREAST_CANCER_*ESR1* _UP | Genes up-regulated in early primary breast tumors expressing *ESR1*  [GeneID=2099] vs the *ESR1*  negative ones. |
|  |  | YANG_BREAST_CANCER_*ESR1* _DN | Genes down-regulated in early primary breast tumors expressing *ESR1*  [GeneID=2099] vs the *ESR1*  negative ones. |
| TERT (Breast) | MSigDb | DAIRKEE_TERT_TARGETS_DN | Genes down-regulated in non-spontaneously immortalizing (NSI) primary breast cancer tumor cultures upon expression of *TERT* [Gene ID=7015] off a retroviral vector. |
|  |  | DAIRKEE_TERT_TARGETS_UP | Genes up-regulated in non-spontaneously immortalizing (NSI) primary breast cancer tumor cultures upon expression of *TERT* [Gene ID=7015] off a retroviral vector. |
| TERT (Tlymphocytes) | MSigDb | ROETH_TERT_TARGETS_DN | Genes down-regulated in T lymphocytes overexpressing *TERT* [Gene ID=7015] off a retrovirus vector. |
|  |  | ROETH_TERT_TARGETS_UP | Genes up-regulated in T lymphocytes overexpressing *TERT* [Gene ID=7015] off a retrovirus vector. |
| TERT (HMEC) | MSigDb | SMITH_TERT_TARGETS_DN | Genes consistently down-regulated in HMEC cells (primary mammary epithelium) upon expression of *TERT* [Gene ID=7015] off a retroviral vector. |
|  |  | SMITH_TERT_TARGETS_UP | Genes consistently up-regulated in HMEC cells (primary mammary epithelium) upon expression of *TERT* [Gene ID=7015] off a retroviral vector. |
| GenIns (Breast) | Habermann JK, Doering J, Hautaniemi S, Roblick UJ, Bundgen NK, et al. (2009) The gene expression signature of genomic instability in breast cancer is an independent predictor of clinical outcome. Int J Cancer 124: 1552-1564. | | |
| GenIns (Bladder) | Jorissen RN, Lipton L, Gibbs P, Chapman M, Desai J, et al. (2008) DNA copy-number alterations underlie gene expression differences between microsatellite stable and unstable colorectal cancers. Clin Cancer Res 14: 8061-8069. | | |
| GenIns (Gastric) | D'Errico M, de Rinaldis E, Blasi MF, Viti V, Falchetti M, et al. (2009) Genome-wide expression profile of sporadic gastric cancers with microsatellite instability. Eur J Cancer 45: 461-469. | | |
| GenIns (ES) | MSigDb | FERREIRA_EWINGS_SARCOMA_UNSTABLE_VS_STABLE_UP | Genes up-regulated in genomically unstable Ewing's sarcoma tumors compared to the stable ones. |
|  |  | FERREIRA_EWINGS_SARCOMA_UNSTABLE_VS_STABLE_DN | Genes down-regulated in genomically unstable Ewing's sarcoma tumors compared to the stable ones. |
| NOTCH1 | MSigDb | NGUYEN_NOTCH1_TARGETS_UP | Genes up-regulated in primary keratinocytes by expression of constantly active NOTCH1 [Gene ID=4851]. |
|  |  | NGUYEN_NOTCH1_TARGETS_DN | Genes down-regulated in primary keratinocytes by expression of constantly active *NOTCH1* [Gene ID=4851]. |
| NFKB | MSigDb | HINATA_NFKB_TARGETS_KERATINOCYTE_UP | Genes up-regulated in primary keratinocytes by expression of p50 (*NFKB1*) and p65 (*RELA*) [Gene ID=4790, 5970] components of NFKB. |
|  |  | HINATA_NFKB_TARGETS_KERATINOCYTE_DN | Genes down-regulated in primary keratinocytes by expression of p50 (*NFKB1*) and p65 (*RELA*) [Gene ID=4790, 5970] components of NFKB. |
| BRCA1 | MSigDb | WELCSH_BRCA1_TARGETS_1_UP | Upregulated by induction of exogenous *BRCA1* in EcR-293 cells |
|  |  | WELCSH_BRCA1_TARGETS_1_DN | Upregulated by induction of exogenous *BRCA1* in EcR-293 cells |
| TGFB1 (Hepatocytes) | MSigDb | COULOUARN_TEMPORAL_TGFB1_SIGNATURE_UP | Late-TGFB1 signature': genes overexpressed in primary hepatocytes at a late phase of *TGFB1* [Gene ID=7040] treatment; is associated with a more invasive phenotype. |
|  |  | COULOUARN_TEMPORAL_TGFB1_SIGNATURE_DN | Early-TGFB1 signature': genes overexpressed in primary hepatocytes at an early phase of *TGFB1* [Gene ID=7040] treatment; is associated with a less invasive phenotype. |
| TGFB1 (Pancreas) | MSigDb | JAZAG_TGFB1_SIGNALING_UP | Genes up-regulated in PANC-1-puro cells (pancreatic cancer) stimulated by *TGF1B* [Gene ID=7040] for 2 h. |
|  |  | JAZAG_TGFB1_SIGNALING_DN | Genes down-regulated in PANC-1-puro cells (pancreatic cancer) stimulated by *TGF1B* [Gene ID=7040] for 2 h. |
| AR (Prostate) | MSigDb | NELSON_RESPONSE_TO_ANDROGEN_UP | Genes up-regulated in LNCaP cells (prostate cancer) in response to synthetic androgen R1881 [PubChem=13766]. |
|  |  | NELSON_RESPONSE_TO_ANDROGEN_DN | Genes down-regulated in LNCaP cells (prostate cancer) in response to synthetic androgen R1881 [PubChem=13766]. |
| AR (Breast) | MSigDb | DOANE_RESPONSE_TO_ANDROGEN_UP | Genes up-regulated in MDA-MB-453 cells (class A ER(-) [Gene ID=2099] breast cancer) after exposure to the androgen R1881 [PubChem=13766]. |
|  |  | DOANE_RESPONSE_TO_ANDROGEN_DN | Genes down-regulated in MDA-MB-453 cells (class A ER(-) [Gene ID=2099] breast cancer) after exposure to the androgen R1881 [PubChem=13766]. |
| ERBB2 (Breast) | MSigDb | LANDIS_ERBB2_BREAST_TUMORS_65_UP | The 65 most significantly changed (p<0.01) genes out of the 324 genes identified by two analytical methods in the mammary tumors induced by transgenic expression of *ERBB2* [Gene ID=2064]. |
|  |  | LANDIS_ERBB2_BREAST_TUMORS_65_DN | The 65 most significantly changed (p<0.01) genes out of the 324 genes identified by two analytical methods in the mammary tumors induced by transgenic expression of *ERBB2* [Gene ID=2064]. |
| ERBB2 (Breast.Her2) | MSigDb | SMID_BREAST_CANCER_ERBB2_UP | Genes up-regulated in the erbb2 subype of breast cancer samples, characterized by higher expression of *ERBB2* [Gene ID=2064]. |
|  |  | SMID_BREAST_CANCER_ERBB2_DN | Genes down-regulated in the erbb2 subype of breast cancer samples, characterized by higher expression of *ERBB2* [Gene ID=2064]. |
| TP53 | MSigDb | KANNAN_TP53_TARGETS_UP | Primary up-regulated targets of *TP53* [GeneID=7157] in the H1299 (lung cancer) cell line. |
|  |  | KANNAN_TP53_TARGETS_DN | Primary down-regulated targets of *TP53* [GeneID=7157] in the H1299 (lung cancer) cell line. |
| EGFR1 | Schulze A, Nicke B, Warne PH, Tomlinson S, Downward J (2004) The transcriptional response to Raf activation is almost completely dependent on Mitogen-activated Protein Kinase Kinase activity and shows a major autocrine component. Mol Biol Cell 15: 3450-3463. | | |
