# Supplementary material for: Breast Tumors with Elevated Expression of 1q Candidate Genes Confer Poor Clinical Outcome and Sensitivity to Ras/PI3K Inhibition
Source: PLoS One. 2013 Oct 17;8(10):e77553. doi: 10.1371/journal.pone.0077553 (PMC3798322; doi:10.1371/journal.pone.0077553)
Supplement: Table S5 — Regression analysis of EXO1 gene expression with pathway activation status in 198 breast tumor samples (GSE7390). (DOCX) [file pone.0077553.s012.docx]

**Table S5.** Regression analysis of *EXO1* gene expression with pathway activation status in 198 breast tumor samples (GSE7390).

|  | **Slope** | **Intercept** | **p-value** | **R-squared** | **Adj.**  **R squared** |
| --- | --- | --- | --- | --- | --- |
| E2F1 | 0.83 | 7.37 | 7.25E-05 | 0.08 | 0.07 |
| MYC | 1.49 | 7.38 | 1.13E-16 | 0.30 | 0.29 |
| E2F3 | 0.29 | 7.35 | 0.214 | 0.01 | 0.00 |
| RAS | 0.71 | 7.39 | 0.001 | 0.05 | 0.05 |
| SRC | -0.37 | 7.38 | 0.049 | 0.02 | 0.01 |
| ESR1 (Vantveer) | -1.22 | 7.42 | 1.65E-20 | 0.36 | 0.35 |
| STAT3 | -0.42 | 7.36 | 0.049 | 0.02 | 0.01 |
| ESR1 (Yang) | -1.12 | 7.45 | 4.40E-15 | 0.27 | 0.27 |
| TERT (Breast) | 0.50 | 7.34 | 0.009 | 0.03 | 0.03 |
| TERT (T lymphocytes) | 0.04 | 7.36 | 0.880 | 0.00 | 0.00 |
| TERT (HMEC) | 1.47 | 7.46 | 2.90E-17 | 0.31 | 0.30 |
| GenIns (Breast) | 0.83 | 7.35 | 8.31E-07 | 0.12 | 0.11 |
| GenIns (Bladder) | 0.76 | 7.37 | 1.83E-06 | 0.11 | 0.11 |
| GenIns (Gastric) | -0.09 | 7.36 | 0.623 | 0.00 | 0.00 |
| GenIns (Ewings Sarcoma) | 1.60 | 7.44 | 7.94E-31 | 0.49 | 0.49 |
| NOTCH1 | -0.50 | 7.38 | 0.015 | 0.03 | 0.02 |
| NFKB | 1.11 | 7.42 | 2.50E-10 | 0.19 | 0.18 |
| TP53 | -1.36 | 7.24 | 2.08E-14 | 0.26 | 0.25 |
| BRCA1 | -0.74 | 7.41 | 0.000 | 0.07 | 0.06 |
| TGFB1 (Hepatocytes) | 1.23 | 7.38 | 6.37E-12 | 0.21 | 0.21 |
| TGFB1 (Pancreas) | 0.85 | 7.36 | 1.66E-05 | 0.09 | 0.09 |
| AR (Prostate) | -0.21 | 7.36 | 0.265 | 0.01 | 0.00 |
| AR (Breast) | -0.70 | 7.36 | 0.001 | 0.05 | 0.05 |
| ERBB2 (Breast) | 0.87 | 7.42 | 2.25E-05 | 0.09 | 0.08 |
| ERBB2 (Breast.HER2) | 0.12 | 7.36 | 0.512 | 0.00 | 0.00 |
| EGFR1 | 1.52 | 7.39 | 1.18E-14 | 0.26 | 0.26 |
