# Supplementary material for: Breast Tumors with Elevated Expression of 1q Candidate Genes Confer Poor Clinical Outcome and Sensitivity to Ras/PI3K Inhibition
Source: PLoS One. 2013 Oct 17;8(10):e77553. doi: 10.1371/journal.pone.0077553 (PMC3798322; doi:10.1371/journal.pone.0077553)
Supplement: Table S6 — Regression analysis of EXO1 gene expression with pathway activation status in 51 breast cancer cell lines (E-TABM-157). (DOCX) [file pone.0077553.s013.docx]

**Table S6.** Regression analysis of *EXO1* gene expression with pathway activation status in 51 breast cancer cell lines (E-TABM-157)

|  | **Slope** | **Intercept** | **p-value** | **R-squared** | **Adj. R squared** |
| --- | --- | --- | --- | --- | --- |
| E2F1 | 0.27 | 6.86 | 0.0126 | 0.12 | 0.10 |
| MYC | 0.24 | 6.90 | 0.0323 | 0.09 | 0.07 |
| E2F3 | -0.15 | 6.90 | 0.2959 | 0.02 | 0.00 |
| RAS | 0.35 | 6.90 | 0.0072 | 0.14 | 0.12 |
| SRC | -0.08 | 6.89 | 0.5272 | 0.01 | -0.01 |
| ESR1 (Vantveer) | -0.31 | 6.85 | 0.0004 | 0.23 | 0.21 |
| STAT3 | 0.24 | 6.87 | 0.1112 | 0.05 | 0.03 |
| ESR1 (Yang) | -0.30 | 6.87 | 0.0005 | 0.22 | 0.20 |
| TERT (Breast) | 0.24 | 6.92 | 0.0286 | 0.09 | 0.08 |
| TERT (T lymphocytes) | 0.00 | 6.89 | 0.9695 | 0.00 | -0.02 |
| TERT (HMEC) | 0.29 | 6.90 | 0.0108 | 0.13 | 0.11 |
| GenIns (Breast) | 0.11 | 6.90 | 0.3015 | 0.02 | 0.00 |
| GenIns (Bladder) | 0.30 | 6.87 | 0.0144 | 0.12 | 0.10 |
| GenIns (Gastric) | 0.24 | 6.92 | 0.0210 | 0.10 | 0.09 |
| GenIns (Ewings Sarcoma) | 0.50 | 6.92 | 0.0000 | 0.41 | 0.40 |
| NOTCH1 | -0.17 | 6.90 | 0.2165 | 0.03 | 0.01 |
| NFKB | 0.41 | 6.90 | 4.91E-05 | 0.29 | 0.27 |
| TP53 | -0.21 | 6.88 | 0.0774 | 0.06 | 0.04 |
| BRCA1 | -0.29 | 6.89 | 0.0327 | 0.09 | 0.07 |
| TGFB1 (Hepatocytes) | 0.27 | 6.91 | 0.0150 | 0.11 | 0.10 |
| TGFB1 (Pancreas) | 0.19 | 6.90 | 0.0944 | 0.06 | 0.04 |
| AR (Prostate) | -0.02 | 6.89 | 0.8774 | 0.00 | -0.02 |
| AR (Breast) | -0.14 | 6.90 | 0.3195 | 0.02 | 0.00 |
| ERBB2 (Breast) | -0.18 | 6.88 | 0.0999 | 0.05 | 0.03 |
| ERBB2 (Breast.HER2) | -0.35 | 6.86 | 0.0045 | 0.15 | 0.14 |
| EGFR1 | 0.41 | 6.92 | 0.0002 | 0.24 | 0.23 |
