# Supplementary material for: Breast Tumors with Elevated Expression of 1q Candidate Genes Confer Poor Clinical Outcome and Sensitivity to Ras/PI3K Inhibition
Source: PLoS One. 2013 Oct 17;8(10):e77553. doi: 10.1371/journal.pone.0077553 (PMC3798322; doi:10.1371/journal.pone.0077553)
Supplement: Table S8 — List of breast cancer signatures used for EXO1 module comparison. (DOCX) [file pone.0077553.s015.docx]

**Table S8.** List of breast cancer signatures used for EXO1 module comparison

| **Signature** | **References** | **Description/Type** | **% of overlap** | **Hypergeometric distribution**  **(P-value)** |
| --- | --- | --- | --- | --- |
| 28-gene signature | [1] | Prognosis | 5.00 | 0.077639 |
| p53-32 | [2] | p53 mutation | 12.00 | 0.000149 |
| TP53 signature | [3] | p53 status to clinical outcome | 40.63 | 0 |
| 3D signature | [4] | Prognosis | 31.82 | 2.64E-12 |
| 64 Gene signature | [5] | Prognosis | 25.49 | 0 |
| Mammaprint | [6] | Prognosis | 12.28 | 3.58E-09 |
| 76 Gene signature | [7] | Prognosis | 6.56 | 0.000118 |
| NCH-70 | [8] | Prognosis | 17.46 | 1.08E-10 |
| GGI-III vs GGI -I | [9] | Grade III | 17.46 | 5.69E-13 |
| IGS (Invasive Gene Signature) | [10] | Prognosis | 0.00 | 0.452945 |
| Wound Response | [11] | Wound response | 60.31 | 0 |
| 12 Gene signature | [12] | Genomic instability | 0.00 | 0.958773 |
| CIN70 | [13] | Genomic instability | 44.44 | 0 |
| Oncotype | [14] | Prognosis | 6.35 | 4.9E-07 |
| 23-gene signature | [15] | Basal | 0.00 | 0.919219 |
| NPI-ES | [16] | Prognosis | 14.29 | 2.66E-13 |

**References**

1. Ma Y, Qian Y, Wei L, Abraham J, Shi X, et al. (2007) Population-based molecular prognosis of breast cancer by transcriptional profiling. ClinCancer Res 13: 2014-2022.

2. Miller LD, Smeds J, George J, Vega VB, Vergara L, et al. (2005) An expression signature for p53 status in human breast cancer predicts mutation status, transcriptional effects, and patient survival. ProcNatlAcadSciUSA %20;102: 13550-13555.

3. Takahashi S, Moriya T, Ishida T, Shibata H, Sasano H, et al. (2008) Prediction of breast cancer prognosis by gene expression profile of TP53 status. Cancer Sci 99: 324-332.

4. Martin KJ, Patrick DR, Bissell MJ, Fournier MV (2008) Prognostic breast cancer signature identified from 3D culture model accurately predicts clinical outcome across independent datasets. PLoSOne %20;3: e2994.

5. Pawitan Y, Bjohle J, Amler L, Borg AL, Egyhazi S, et al. (2005) Gene expression profiling spares early breast cancer patients from adjuvant therapy: derived and validated in two population-based cohorts. Breast Cancer Res 7: R953-R964.

6. van 't Veer LJ, Dai H, van de Vijver MJ, He YD, Hart AA, et al. (2002) Gene expression profiling predicts clinical outcome of breast cancer. Nature 415: 530-536.

7. Wang Y, Klijn JG, Zhang Y, Sieuwerts AM, Look MP, et al. (2005) Gene-expression profiles to predict distant metastasis of lymph-node-negative primary breast cancer. Lancet %19-25;365: 671-679.

8. Naderi A, Teschendorff AE, Barbosa-Morais NL, Pinder SE, Green AR, et al. (2007) A gene-expression signature to predict survival in breast cancer across independent data sets. Oncogene 26: 1507-1516.

9. Sotiriou C, Wirapati P, Loi S, Harris A, Fox S, et al. (2006) Gene expression profiling in breast cancer: understanding the molecular basis of histologic grade to improve prognosis. JNatlCancer Inst 98: 262-272.

10. Liu R, Wang X, Chen GY, Dalerba P, Gurney A, et al. (2007) The prognostic role of a gene signature from tumorigenic breast-cancer cells. NEnglJMed 356: 217-226.

11. Chang HY, Sneddon JB, Alizadeh AA, Sood R, West RB, et al. (2004) Gene expression signature of fibroblast serum response predicts human cancer progression: similarities between tumors and wounds. PLoSBiol 2: E7.

12. Habermann JK, Doering J, Hautaniemi S, Roblick UJ, Bundgen NK, et al. (2009) The gene expression signature of genomic instability in breast cancer is an independent predictor of clinical outcome. IntJCancer 124: 1552-1564.

13. Carter SL, Eklund AC, Kohane IS, Harris LN, Szallasi Z (2006) A signature of chromosomal instability inferred from gene expression profiles predicts clinical outcome in multiple human cancers. NatGenet 38: 1043-1048.

14. Paik S, Shak S, Tang G, Kim C, Baker J, et al. (2004) A multigene assay to predict recurrence of tamoxifen-treated, node-negative breast cancer. NEnglJMed 351: 2817-2826.

15. Lin Y, Lin S, Watson M, Trinkaus KM, Kuo S, et al. (2010) A gene expression signature that predicts the therapeutic response of the basal-like breast cancer to neoadjuvant chemotherapy. Breast Cancer ResTreat 123: 691-699.

16. Yu K, Lee CH, Tan PH, Hong GS, Wee SB, et al. (2004) A molecular signature of the Nottingham prognostic index in breast cancer. Cancer Res 64: 2962-2968.
